# Ethical and practical considerations for interventional HIV cure-related research at the end-of-life: A qualitative study with key stakeholders in the United States

**DOI:** 10.1371/journal.pone.0254148

**Published:** 2021-07-16

**Authors:** John Kanazawa, Sara Gianella, Susanna Concha-Garcia, Jeff Taylor, Andy Kaytes, Christopher Christensen, Hursch Patel, Samuel Ndukwe, Stephen Rawlings, Steven Hendrickx, Susan Little, Brandon Brown, Davey Smith, Karine Dubé

**Affiliations:** 1 Gillings School of Global Public Health, University of North Carolina at Chapel Hill, Chapel Hill, North Carolina, United States of America; 2 Division of Infectious Diseases and Global Public Health, Department of Medicine, University of California San Diego, La Jolla, California, United States of America; 3 HIV Neurobehavioral Research Program (HNRP), California NeuroAIDS Tissue Network, University of California San Diego, San Diego, California, United States of America; 4 AVRC Community Advisory Board, University of California San Diego, San Diego, California, United States of America; 5 HIV + Aging Research Project–Palm Springs (HARP-PS), Palm Springs, California, United States of America; 6 AntiViral Research Center (AVRC), University of California at San Diego, San Diego, California, United States of America; 7 Department of Social Medicine, Population and Public Health, Center for Healthy Communities, University of California, Riverside, Riverside, California, United States of America; Emory University, UNITED STATES

## Abstract

**Background:**

A unique window of opportunity currently exists to generate ethical and practical considerations presented by interventional HIV cure-related research at the end-of-life (EOL). Because participants would enroll in these studies for almost completely altruistic reasons, they are owed the highest ethical standards, safeguards, and protections. This qualitative empirical ethics study sought to identify ethical and practical considerations for interventional HIV cure-related research at the EOL.

**Methods and findings:**

We conducted 20 in-depth interviews and three virtual focus groups (N = 36) with four key stakeholder groups in the United States: 1) bioethicists, 2) people with HIV, 3) HIV care providers, and 4) HIV cure researchers. This study produced six key themes to guide the ethical implementation of interventional HIV cure-related research at the EOL: 1) all stakeholder groups supported this research conditioned upon a clearly delineated respect for participant contribution and autonomy, participant understanding and comprehension of the risks associated with the specific intervention(s) to be tested, and broad community support for testing of the proposed intervention(s); 2) to ensure acceptable benefit-risk profiles, researchers should focus on limiting the risks of unintended effects and minimizing undue pain and suffering at the EOL; 3) only well-vetted interventions that are supported by solid pre-clinical data should be tested in the EOL translational research model; 4) the informed consent process must be robust and include process consent; 5) research protocols should be flexible and adopt a patient/participant centered approach to minimize burdens and ensure their overall comfort and safety; and 6) a participant’s next-of-kin/loved ones should be a major focus of EOL research but only if the participant consents to such involvement.

**Conclusions:**

To our knowledge, this empirical ethics study generated the first ethical and practical considerations for interventional HIV cure-related research at the EOL. The ethical complexities of such research must be considered now. We must navigate this ethical conundrum so that we are good stewards of the participants’ extremely altruistic gifts by maximizing the impact and social value of this research. We hope that this study will serve as the foundation for future research and discussion on this topic.

## Introduction

With over 250 completed or active biomedical studies globally, the search for an HIV cure is intensifying [1]. When referring to a cure for HIV, we are referring to either a cure which completely eliminates HIV from the body or a regimen which induces long-term viral suppression free of antiretroviral therapy (ART) [2]. Until recently, otherwise healthy people with HIV (PWH) have been the usual sample population for most HIV cure-related studies [3, 4]. Now, researchers are including PWH with non-AIDS terminal illnesses at the end-of-life (EOL) in this research to measure the latent HIV reservoir following rapid research autopsy [3, 5, 6]. Due to the large tissue samples required and the rapid degeneration of the HIV genome after death, rapid research autopsies on PWH at the EOL is currently the only method for accurately assessing the HIV reservoir in deep tissue compartments [6–8]. Further, enrolling PWH at the end of their life allows a close follow-up during a limited period of time to collect detailed clinical data until the very end (days and hours before death). This granularity is crucial to correctly interpret the HIV reservoir measures. For example, if a participant interrupts ART at the end of life (even just a few days before death), this will affect the outcomes.

To date, these studies have been observational, but researchers will soon begin testing interventions in this population. The University of California San Diego (UCSD) enrolls PWH who are terminally ill and have a prognosis of six months or less, as well as chronically ill PWH with concomitant co-morbidities who are nearing the EOL, into one such observational HIV cure-related study known as “The Last Gift” [3]. Participants in this study agree to donate blood and body fluid samples while alive and to donate their entire bodies for rapid research autopsy at the time of death [3]. The Last Gift study serves as the anticipated paradigm for which biomedical researchers are expected to begin testing interventions in PWH nearing the EOL, thus necessitating the proactive investigation of the ethical and practical considerations for implementing such studies.

As demonstrated by our previous research, a six-pronged rationale exists for conducting HIV cure-related research in PWH at the EOL: 1) these studies offer no reasonable expectation of direct clinical benefits, 2) aging PWH have expressed a manifest desire to advance the search towards an HIV cure [5], 3) aging and terminally ill PWH have minimal opportunities to participate in HIV research, 4) higher risks for research participation may be acceptable for PWH at the EOL, 5) rapid research autopsy is possible in this population through body donation, and 6) a unique opportunity is presented to create a novel translational model to test interventions on human participants [3]. While the potential scientific knowledge to be gained is vast by testing interventions in PWH at the EOL and the social value of this research is enormous [9, 10], it is also rife with ethical concerns [8, 11, 12]. This manuscript builds upon our prior work which detailed ethical considerations for observational HIV cure-related research at the EOL [3, 13–15].

Though interventional HIV cure-related research at the EOL is not yet happening, it seems inevitable that it will. We need robust ethical safeguards in place before this eventuality occurs [16]. A unique window of opportunity currently exists to generate ethical and practical considerations presented by interventional EOL HIV cure-related research. By interventional HIV cure-related research, we mean testing latency-reversing agents (LRAs), immune-based interventions, cell and gene therapy (CGT) approaches, or other HIV cure-related strategies in PWH nearing the EOL. Further, analytical treatment interruptions (ATIs), or the purposeful stopping of ART, are necessary in this research to validate any resultant effect of the interventions’ ability to keep HIV suppressed for an extended period of time in the absence of ART [17, 18]. Empirical work is needed to shape future protocols that seek to employ novel interventions in the EOL translational model. In this research, PWH approaching the EOL would undergo interventions, not in the hope of extending their own lives, but with the sole intent of advancing HIV cure-related research [3, 7]. We therefore endeavored to identify core ethical and practical considerations relevant to interventional HIV cure-related research at the EOL by conducting qualitative, in-depth interviews and focus groups with four key stakeholder groups in the United States: 1) bioethicists, 2) PWH, 3) HIV care providers, and 4) HIV cure researchers. Through this multi-disciplinary approach, we sought to begin this essential and timely conversation around the ethics of testing interventions in PWH nearing the EOL.

## Methods

### Study setting and participants

Our informants were selected based on prior exposure to, and knowledge of, HIV cure-related research at the EOL from diverse groups, such as academic institutions, community advisory boards (CABs), community-based organizations, funding agencies, and HIV clinics. Participants for our 20 in-depth interviews were recruited from the four above-mentioned key stakeholder groups (Table 1), and participants for our three virtual focus groups were selected from PWH (i.e., community members) (Table 2) using a purposive and non-probabilistic sampling technique [19]. This sampling technique was employed to gain triangulated perspectives because the ethics of conducting interventional HIV cure-related research at the EOL is a novel topic.

**Table 1:** Demographic characteristics of key informant interview participants (United States, 2020).

Participant Number	Sex	Race/Ethnicity	Informant Type
101	Male	Caucasian/non-Hispanic	Researcher
102	Male	Caucasian/non-Hispanic	Bioethicist
103	Female	Caucasian/non-Hispanic	Researcher
104	Male	Caucasian/non-Hispanic	Community member
105	Male	Caucasian/non-Hispanic	Researcher
106	Male	Caucasian/non-Hispanic	Researcher
107	Male	Caucasian/non-Hispanic	Researcher
108	Female	American Indian/Hispanic	Researcher
109	Male	Caucasian/non-Hispanic	Researcher
110	Male	Caucasian/non-Hispanic	Researcher
111	Female	Asian	HIV clinician
112	Male	Caucasian/non-Hispanic	Researcher
113	Male	Caucasian/non-Hispanic	Researcher
114	Male	Caucasian/Hispanic	HIV clinician
115	Female	Caucasian/non-Hispanic	HIV clinician
116	Male	Caucasian/non-Hispanic	Researcher
117	Male	Caucasian/non-Hispanic	Researcher
118	Male	Caucasian/non-Hispanic	Researcher
119	Female	Caucasian/non-Hispanic	Researcher
120	Female	Caucasian/non-Hispanic	HIV clinician

**Table 2:** Demographic characteristics of focus group participants (Southern California, 2020).

	FG-1	FG-2	FG-3	Total	Percent
*n*	6	3	7	16	
Gender					
Male	4	2	5	11	68.8
Female	2	1	2	5	31.3
Transgender (male to female)	0	0	0	0	0
Transgender (female to male)	0	0	0	0	0
Gender queer/non-binary	0	0	0	0	0
Did not specify	0	0	0	0	0
Age (Median: 58; Range: 47–78)					
40–49	0	1	1	2	12.5
50–59	2	1	2	5	31.3
60–69	1	1	2	4	25.0
70–79	0	0	1	1	6.3
Did not specify	3	0	1	4	25.0
Race/Ethnicity*					
Caucasian/White	5	2	3	10	62.5
Black/African-American	0	1	4	5	31.3
Hispanic/Latino Descent	0	0	1	1	6.3
American Indian/Alaska Native	0	0	1	1	6.3
Native Hawaiian/ Other Pacific Islander	0	0	0	0	0
Asian/Asian Descent	0	0	0	0	0
Other	0	0	0	0	0
Did not specify	1	0	0	1	6.3

*Some participants identified with more than one race/ethnicity.

Normative ethics, or how one *should* morally act, is contextualized by empirical ethics; the latter evaluates people’s thoughts about what should ethically happen in real-world settings [20]. Because of the formative nature of the topic at hand and a scarcity of relevant data, we used a qualitative approach for this study [21, 22] to capture the rich, nuanced empirical ethics considerations from our informants by way of in-depth interviews and focus groups [23].

### Participant recruitment

#### Key informant interviews

Potential informants were identified by the study’s principal investigator (K.D.) in collaboration with community co-investigators (J.T., C.C., and A.K.) and an external Scientific Advisory Board. Email invitations were then sent to the identified potential informants. Interviews were scheduled with informants upon acceptance of our invitation (response rate: 66%), and copies of the informed consent form, the demographic questionnaire, and the interview guide were provided to them.

#### Virtual focus groups

We reached out to two Southern California community groups, the AntiViral Research Center (AVRC) in San Diego, CA, and the HIV + Aging Research Project–Palm Springs, CA (HARP-PS), that have been actively advising the Last Gift Study and collaborating with members of our study team since 2017 to recruit community members for our virtual focus groups. We approached these groups and recruited these participants because of their high level of HIV literacy and their previous exposure to HIV cure-related research at the EOL. Each community group’s leaders and coordinators assisted with arranging and scheduling of the virtual focus groups.

#### Data collection

The informed consent form was sent to all participants in advance of their interview or focus group. All interviews and focus groups were conducted and recorded in the English language on a virtual conferencing platform that was compliant with the Health Insurance Portability and Accountability Act (HIPAA). Verbal consent was obtained and recorded from all interview participants. Data security measures were sent to all focus group participants prior to the focus groups, and written consent was obtained from all focus group participants. An institutional review board (IRB)-approved interview guide was used to facilitate all interviews, and community-friendly, IRB-approved slides were used to guide virtual focus group conversations. A copy of our guide can be found in Table 3.

**Table 3:** IRB-approved interview guide and focus group question route: Ethical and practical considerations for interventional HIV cure-related research at the EOL.

**Testing Interventions in People with HIV at the EOL**
• Do you think it would be ethical to test interventions in PWH who are approaching the EOL? Why/why not?
• What would be the benefits of testing interventions in PWH who are approaching the EOL?
• What would be the risks of testing interventions in PWH who are approaching the EOL?
• How do we ensure this research remains within acceptable benefit/risk parameters?
• How do we minimize burdens to study participants at the EOL?
• What do you think would be ‘too much risk’ for an intervention at the EOL?
• What would be some of the considerations for next-of-kin/loved ones when testing interventions at the EOL?
○ Should the next-of-kin/loved ones also provide informed consent?
**Considerations for Specific HIV Cure-Related Research Strategies**
• Do you think we should test latency-reversing agents in the EOL translational research model? Why/why not?
○ Which latency-reversing agents may be best suited? Why is that?
○ What safeguards would need to be in place for testing latency-reversing agents?
• Do you think we should test immune-based interventions in the EOL translational research model? Why/why not?
○ Which immune-based interventions may be best suited? Why is that?
○ What safeguards would need to be in place for testing immune-based interventions?
• Do you think we should test cell and gene approaches in the EOL translational research model? Why/why not?
○ Which cell and gene approaches may be best suited? Why is that?
○ What safeguards would need to be in place for testing cell and gene approaches?
• Do you think we should perform stem cell transplants in PWH at the EOL? Why/why not?
• Do you think we should test combination approaches in PHW at the EOL? Why/why not?
○ If yes, which combination approaches may be best suited? Why is that?
○ If yes, what safeguards would need to be in place for testing combination approaches?
• Do you think we should perform analytical treatment interruptions in the EOL translational research model? Why/why not?
○ What safeguards would need to be in place for testing combination approaches?
**Additional Considerations**
• In clinical research, death is classified as a serious adverse event (SAE). How should research teams deal with the SAE issue in EOL HIV cure-related research? Do you think the type of research would make SAE ascertainment difficult? If so, in what way(s)?
• Do you think interventions should be tested at the EOL with individuals with concomitant conditions (e.g. HIV and cancer)?
**Wrap Up and Closing**
• Would you like to add anything or make additional comments?

Each interview and focus group was conducted by two members of our research team (K.D. and J.K.), each of which kept detailed field notes. Compensation in the form of a $20 Visa gift card was provided to community members; no compensation was provided to informants representing academic institutions or funding agencies.

#### Data analysis

All audio files were saved on a secure drive with access limited to two members of the research team (J.K. and K.D.). Each file was uploaded by a member of the research team (J.K.) to an encrypted website for verbatim transcription immediately following each interview and focus group. Each transcript was reviewed by a member of the research team (J.K.) for completeness and accuracy by comparing the audio recording to the transcript. Participants were not invited to comment, correct, or provide feedback on the results of their interviews or focus groups. To ensure protection of confidential information, all documents and transcripts were de-identified, and all audio recordings were destroyed after quality assessment of the related transcript.

To adequately assess the emergent data obtained from this novel and exploratory study, we employed thematic content analysis via inductive reasoning as our methodological approach [24]. Saturation, the point when no new information or themes are observed in the data [25], was likely not reached after 20 in-depth interviews and 3 virtual focus groups.

We employed the consolidated criteria for reporting qualitative research (COREQ) checklist [26] and used a high degree of fidelity to our interview guide during both the interviews and focus groups. This allowed us to organize the data by collating all responses for each question of the guide, regardless of whether the data was generated from interviews or focus groups, into a single, master document. Responses were organized by informant types, allowing us to review the range and richness of responses obtained. After compilation of the de-identified answers into a master document, two members of our research team (J.K. and K.D.) manually coded the data into emergent themes and sub-themes using the inductive approach discussed above. By using this method, we ensured no difference in themes elicited by method of data generation. The two coders (J.K and K.D.) began by independently ascribing tags to the data. The two data sets were then compared, and initial themes were elicited. Key themes and sub-themes were then derived by the primary coder (K.D.), who also generated the initial code book and extracted salient quotes relative to considerations for conducting intervention HIV cure-related research at the EOL. After reviewing the primary coder’s assessment, refinements were made as necessary by the secondary coder (J.K.) and quotations were organized into narratives and tables. Discrepancies that arose were resolved by discussion and consensus to ensure consistency, validity, and reliability in the interpretation of the data. The most illustrative quotations associated with major themes can be found in the results section. Supplementary quotations are included in the (S1 Table).

#### Ethics statement

The Institutional Review Board of the University of North Carolina at Chapel Hill approved this empirical research ethics study (study #: 19–0522). All interview and focus group participants included in this study provided informed consent to participate.

## Results

We interviewed 14 cisgender men and 6 cisgender women, most of whom were Caucasian/non-Hispanic (Table 1). We recruited 14 biomedical HIV cure researchers, 4 HIV clinicians, 1 community member, and 1 bioethicist. Informants for our interviews worked in the field of HIV for a mean of 22 years (SD: 10.1 years), and in the field of HIV cure-related research for a mean of 8.8 years (SD: 7.9 years). Our virtual focus groups were comprised of community members and included 11 cisgender men and 5 cisgender women with HIV aged 47–78 years (Table 2). Of these, 10 were Caucasian/White, 5 were African-American/Black, and 1 was American Indian/Alaskan Native and of Hispanic/Latino descent.

Our study found the following key themes to guide the implementation of interventional HIV cure-research at the EOL:

1. All key stakeholder groups supported this research conditioned upon a clearly delineated respect for participant contribution and autonomy, participant understanding and comprehension of the risks associated with the specific intervention(s) to be tested, and broad community support for testing of the proposed intervention(s).
2. To ensure favorable benefit-risk ratios, researchers should focus on limiting the risks of unintended effects and minimizing undue pain and suffering.
3. Only well-vetted interventions that are supported by solid pre-clinical data should be tested in the EOL translational research model, though divergent views were given on the specific interventions best suited for testing at the EOL.
4. The informed consent process must be robust and include process consent.
5. Research protocols should be flexible and adopt a patient/participant centered approach to minimize burdens to participants and ensure their overall comfort and safety.
6. NOK/loved ones generally should be a major focus of EOL research if and only if the participant consents to such involvement, even though tension may exist between NOK/loved ones involvement in research and a participant’s autonomy.

### Perceptions of testing interventions in the EOL translational research model

#### Ethicality of interventional HIV cure-related research at the EOL

We asked informants if they thought it would be ethical to test interventions in PWH at the EOL. Generally, informants reacted favorably. A bioethicist noted:

*I don’t think it’s ethical*, *period*, *as an absolute*. *I think that it can be ethical*. *I think it*. . . *actually I’ll even go a step further*, *I would say it would be unethical to not try and find a way to design these studies in an ethical fashion*. *I’ve been convinced from what I’ve heard so far that it’s worth doing*, *but we need to find a way to do it ethically*. *So the answer is*, *it should be ethical*.–#102

Researchers noted that HIV cure-related research at the EOL could allow for the testing of higher-risk interventions that would not be ethically allowed to test in otherwise healthy PWH (Researchers #107 and #116). One researcher stated:

*We all die*. *So not performing research on dying people seems like we’re just leaving out [a large segment of the] population*.–Researcher #105

Community members overwhelmingly responded in the affirmative towards testing interventions in PWH at the EOL. Referring to the Last Gift study, one community member made the statement that:

*Many of them [the participants]*, *for many years*, *have been denied the opportunity to participate in research because they’re HIV-positive or because they’re older*. *So this is … not just the Last Gift*, *it’s really the last chance for these participants [to] pay back [the community because] they benefited from [prior HIV research]*.–Community member #104

Community members also stressed the fact that it is a personal choice to participate in interventional HIV cure-related research at the EOL and that each participant should be allowed to make that choice when fully informed of the risks (FG-1 participant). Likewise, HIV clinicians and researchers espoused the same view:

*I think so*. *I think*, *going back to autonomy and consent*, *the individual really must be aware of what is being talked about*, *of the risks that are going to be involved*. *If an individual wants to give of their time of themselves by participating in such a study*, *then it would be*, *I think*, *unethical to withhold their right to participate*.–HIV clinician #114

Informants recognized that ethicality is dependent on the intervention being tested and on the participant’s comprehension of the intervention’s associated risks:

*I mean I think it’s difficult to answer that question because I think it depends on the intervention*. *Again*, *whether or not it could cause suffering*, *whether it could shorten lifespan*, *all of these things potentially will differ between each participant in terms of what matters to them*. *Some persons may want to live as long as they possibly can*, *other persons may want to experience no suffering at the end of their life*. *So I think it’s just hard to answer that question because it really is a personal matter*, *and really does depend most on what matters to that individual participant*.–HIV clinician #111

*Again*, *as long as the consent process is very clear*, *and they know the risks that they are getting into with it*. *That to me is an unbelievably big decision for someone to make*. *And to really understand all of the implications*, *and what that could look like for them is really important*.–HIV clinician #115

In addition to the foregoing, researchers noted that this research is also subject to community ethical standards and that research teams should “be attentive and listen to what the community is ready to do” (#110 and #118). Researchers appreciated the gravity of a participant’s willingness to participate in interventional HIV cure-related research at the EOL:

*If someone makes such a generous gift at the end of life, that you need to do everything possible to make sure that that gift has the most impact it possibly can. You would not want someone to waste that contribution on something that’s not likely to move the needle, and you would want to make sure that the very best people are accessing those tissues and answering the question. You would want to make sure that it’s an important question because it’s such a charged thing. From the participant’s side of things, for many people, it’s a very profound thing that they’re. . . giving back. It’s a very meaningful thing to them. The researcher wants to honor that in as best a way that they can*.

–Researcher #113

Overall, our informants expressed support for testing HIV cure-related interventions at the EOL. They also explicitly stated, however, that their support was conditional, namely on participant autonomy, participant comprehension and understanding of the risks of a specific intervention to be tested, community support for the proposed intervention, and respect for the participant’s gift at the EOL.

#### Perceived benefits of testing interventions at the EOL

We asked informants about the perceived benefits of testing interventions at the EOL. Societal benefits, such as the knowledge to be gained and the potential for developing a cure, emerged as points of convergence:

*But if that intervention could teach us something so important about how to cure HIV*, *that it leads to a cure for millions*, *there’s a real reason to pursue testing that intervention*.–Researcher #105

*I would imagine any potential curative research would have tremendous benefit for society*. *We’ve been working for decades*, *at this point*, *to understand and prevent this epidemic*, *reverse it*. *This would be part of that*. *I do feel like we’re going in the right direction in that regard*.–HIV clinician #120

*The advantage*, *obviously of these end-of-life studies is that if these people consent to give their body after they pass away*, *obviously you will have an unlimited amount of material to actually look at the effect of the intervention*, *which is unique*.–Researcher #117

Informants also recognized the novelty of testing HIV cure-related interventions in an EOL population. For some interventions, there is currently “no other feasible way” to test the interventions (Researcher #119) in otherwise healthy volunteers because of the inherent clinical risks of the interventions and the effectiveness of current ART which create a high safety threshold for testing these interventions in otherwise healthy PWH:

*So the threshold to increase risk to anybody with HIV who’s well controlled*, *the threshold*, *to expose them to increased risk is actually quite high*. *You would need to have a real reason to expose them to risk for benefit*. *In end of life*, *so if that risk includes death*, *you have much less willingness to take a 35-year-old who has a long life ahead of them*, *and potentially expose them to death to cure disease that otherwise they could live with for a long time*.–Researcher #105

Likewise, HIV clinicians and researchers recognized that, by testing interventions in an EOL population, the risk of long-term harm to their participants would be diminished:

*So I think the biggest benefit is that there’s*. . . *there is a decreased likelihood that you’re going to do irreparable harm*, *that they will have to live with for a very long time*.–HIV clinician #111

*There may be some types of interventions that could have potential for long-term side effects*. *So*, *well*, *if we know that you’re going to die of some other disease in the next month*, *is that really a concern if you may get a cancer in 10 years*? *It would be for a healthy volunteer*, *but it may not be that much of a risk at all for someone who knows that they’re at the end of their life*.–Researcher #113

Though our informants recognized that participants were highly unlikely to receive any clinical benefits from participation in this research (Researchers #106 and #110), they recognized the participant’s intangible senses of personal fulfillment or satisfaction as perceived personal psychosocial benefits to be gained:

*[P]ersonally*, *of course*, *as we’ve discussed*, *giving that sense of purpose*, *a chance to give back*, *a chance to benefit*. *I think especially for long-term survivors*, *they’ve been through so much in terms of how they’ve suffered*, *and they’ve seen their communities and loved ones suffer through the disease*, *[that] being able to make an impact*, *a lasting impact*, *when they really haven’t been able to in recent years is really important*.–Community member #104

*[T]he altruism that somebody could potentially transition*, *thinking*, *"I am a part of the cure*. *I am a part of the future*.*" All of us*, *I think*, *want to make*, *at least*, *a bit of an imprint on our world*. *Doing it in this way could be very life-affirming for them*.–HIV clinician #120

*I think that one of the benefits is also for the person itself*, *that they feel they are contributing to the society while they are at the end of life*.–Researcher #103

When asked to describe potential benefits of testing HIV cure-related interventions at the EOL, our informants pointed to two distinct groups of benefits: societal and personal. The societal benefits revolved around the knowledge to be gained and the advancement towards an HIV cure. The personal benefits that inure to the participants are not quantifiable clinical benefits but are the intangible psychosocial benefits of altruism and personal fulfillment.

#### Perceived risks of testing interventions at the EOL

The most cited perceived risks of interventional HIV cure-related research at the EOL were the possibility of decreased quality of life (QOL), increased suffering, and hastening of death. Community members, HIV clinicians, and researchers alike were concerned about increasing suffering and decreasing the QOL in PWH at the EOL:

*We don’t want to hurt anybody*, *so I think the idea that we’ve all been trained forever first to do no harm*. *A participant can say*, *"I’m willing to suffer this much*. *I’m willing to do this much that might hurt me*,*" but at the end of the day*, *we really don’t want to do that*.–Researcher #119

*[S]o just thinking from a clinician’s standpoint*, *what I would find acceptable and unacceptable for the people that I care for*, *the thing that I would be most concerned about is if the intervention could potentially be painful or could harm them in any way [and] whether it [could be] … even exploitative*. *Because if indeed we’re going to be performing research at the end of life*, *I would want my primary goal to make sure that whatever is the remainder would be a positive experience for the person*, *the participant*.–HIV clinician #111

*I think the quality of the end of your life is extremely important*. *The limit I would put is really how much pain that you will cause to these people*.–Researcher #117

Likewise, informants were also concerned that any intervention may accelerate or hasten the death of the participant (HIV clinician #114):

*So*, *when do you start asking people to give of themselves that could potentially shorten their lives*? *What’s that appropriate timeframe*? *And I think we all struggle with that*.–HIV clinician #115

HIV clinicians and researchers also warned of potential scientific risks, such as generating knowledge of little or no value (HIV clinician #114) and/or non-generalizable findings due to small numbers (Researcher #116). They were also concerned with the resultant public perceptions of this research or “societal misunderstanding that you’re experimenting [on] people and causing harm” (Researcher 119):

*Even though I’m absolutely confident that it would be in the informed consent that you could conceivably cause harm including death*, *accelerated death*. *All of these people are end of life and expected to die*. *If that happened*, *I could see it causing significant grief among next of kin*, *among friends*, *family*, *and investigators…*. *even if there was a modest amount of pain and suffering*, *I think the societal blowback could be very substantial*, *and it could end up just ending the program*.–Researcher #119

The bioethicist raised concerns related to the participant’s personal relationships and family dynamics:

*So the risks are … that somebody could*, *either consciously or unconsciously*, *choose to be in the study when it isn’t really right for them psychologically [or] personally*, *in terms of their relationship to their family and friends*.–Bioethicist #102

Disruptions to participants’ and caregivers’ schedules also concerned HIV clinicians and researchers (HIV clinician #115 and Researcher #118).

The perceived risks of testing HIV cure-related interventions at the EOL centered around the participant’s potentially increased suffering, decreased QOL, and accelerated death. Informants also noted the scientific risks of non-generalizable data, as well as the potential for negative public perceptions. The bioethicist pointed out the potential for interpersonal risks.

#### Ways to ensure acceptable benefit/risk profiles for testing interventions at the EOL

We asked informants to suggest ways of ensuring an acceptable benefit/risk profile for interventional HIV cure-related research at the EOL. Researchers strongly supported only testing interventions that had solid pre-clinical data and had been vetted at multiple levels:

*I mean, you could argue that the normal risk-benefit analysis is perturbed by the fact this person is going to die. So, I don’t think you can do the normal risk-benefit analysis. I think you have to just make sure that it’s not something that is a scientific form of euthanasia. It has to be at least proven, in some way, in animals, still, to make sure that there’s no significant, immediate risks to life, due to the treatment*.

–Researcher #118

Informants further suggested multi-disciplinary research teams, including bioethicists and socio-behavioral scientists (Community member #104), who engage in open communication with the community (HIV clinician # 111 and Researcher #119) as another way to ensure an acceptable benefit/risk profile. Additionally, informants from each of our four key stakeholder groups recommended the informed consent process be “clearly explained to the participant” (HIV clinician #114) and include “process consent,” an iterative, continuous consent process involving members of the research team and the participant [27]:

*When circumstances or plans change*, *when the individuals’ reactions or responses change*. *At every step of the way we have to readdress with them*.–Bioethicist #102

*You may want to consider … some supplementary consent*. *That*, *you sign an initial consent to participate in the study but then periodically*, *[re]consent [and] sign … again*.–FG-3 participant

Our informants suggested testing only well-vetted interventions with solid pre-clinical data and convening multi-disciplinary research teams as ways to ensure acceptable benefit/risk profiles for testing interventions at the EOL. Likewise, they converged on participants’ clear understanding of risks and benefits and pointed out that the informed consent process should include process consent.

#### Perceptions of ‘too much’ and/or unacceptable risk at the EOL

We asked informants to describe a situation when testing HIV cure-related interventions at the EOL would be untenable. The bioethicist, community members, and researchers all responded that the determination of what comprised “too much risk” should first be left to the participant’s discretion:

*That assessment is first up to the person who is accepting a burden of being part of this research study…*. *They get first crack at the decision that if the risk is too much…*. *Risk is a combination of what the severity of the consequences are*, *and the probability that that will actually occur…*. *But we have verified that they’ve understood what those risks are*. *So that they are the one getting to make a decision about whether they want to carry that burden or not*.–Bioethicist #102

*Too much risk is whatever the patient is not willing to take*.–Researcher #103

Many informants cited “undue pain and suffering” (Community member #104) as constituting too much risk:

*I think we probably even want to be even more cautious and really put limits that I think should be first the pain*. *They’re already suffering*, *not only physically*, *but psychologically and these are obviously difficult times for them and the people that are close to*.–Researcher #117

*I’m not going to cause, intentionally, pain and suffering. I think if there are interventions that come with some degree of discomfort, and I can mitigate that discomfort so that there isn’t undue pain and suffering, that is something I am willing to discuss. I can’t in good conscience go through with something that is going to cause significant pain and suffering that I can’t do anything about. It doesn’t compute*.

–Researcher #119

Rapidly hastening death also constituted unacceptable risk to most informants:

*I think a no go would be*, *"We do this*, *and you could die in a day or two*.*"*–HIV clinician #115

*That’s one line, we’re not going to be God. We’re not going to introduce an intervention that’s going to terminate somebody’s life or put them at a risk that that would be an immediate thing*.

–Researcher #116

Some HIV clinicians and researchers also pointed out that any intervention with the potential to cause unintended effects that lead to prolonged pain and suffering would be a non-starter, particularly if the intervention also extended the participant’s life (HIV clinician #115 and Researcher #107). One researcher also pointed out that “asking a participant to undergo all of this without getting much in return for science” would be unacceptable (#109).

In sum, the determination of when an intervention would pose too much risk at the EOL is one that should first be left to the participant. Research teams should also seek to ensure that interventions minimize the risks of undue pain and suffering, rapidly accelerating death, or unintended effects.

#### Ways to minimize participant burdens at the EOL

When asked for ways to minimize burdens to participants at the EOL, the bioethicist succinctly summarized the overall perception of all our informants:

*We should look for every opportunity we can to minimize those burdens*. *If you can restrict the number of blood draws*, *if you can restrict the number of pills somebody has to take or restrict the number of times you interview them*.–Bioethicist #102

Informants from all four groups stated, either implicitly or explicitly, that studies should be designed in such a way as to allow for “flexibility” (FG-2 participant). Further, research protocols should remain adaptable to each participant, even if it means a greater burden for the research staff (Researcher #107):

*I think when creating the study I think it’s just going to be really crucial to find ways to adapt*. *And I think we do that a lot with research*, *but I think that especially if you’re looking at end of life individuals*, *they might have some other events and I think it’s just really important to understand that your research has to be a little more flexible and adaptive*.–Researcher #107

*I suspect this is going to be patient-specific or participant-specific*. *[I]t’ll have to be tailored to each individual participant*, *well how many blood draws they will feel is a burden*, *and how many hospital visits they will feel are a burden*.–Researcher #112

HIV clinicians and researchers called for protocols to be designed to maximize the scientific benefit of study visits and procedures like blood draws (HIV clinician #115 and Researcher #109). They also suggested the use of “proxy measures, such as urine or hair clippings” that could provide valuable information to the study team while causing minimal discomfort to the participant at the EOL (#109).

Community members, HIV clinicians, and researchers all favored research teams going to the participants for study visits as a way to minimize burdens:

*You go to them. I’ve actually always been a proponent of this in clinical care, but we can’t get it done because the hospital doesn’t like paying for stuff like this. But I think if you go to them, then that can significantly minimize the burden. Have a mobile research team, with a phlebotomist, and a research nurse, who goes out to the study participants in whatever environment they’re comfortable in*.

–HIV clinician #111

All informant groups recognized blood draws as being particularly burdensome for PWH at the EOL. Community members and researchers explicitly stated that it should not be necessary for a participant to travel anywhere for a simple blood draw; instead, these procedures should be conducted at the participant’s residence (FG-1 participant and Researcher #112) or by using a home-based blood collection device. Community members also suggested minimizing the number of “sticks” and favored installation of a central-line or port (Community member #104 and FG-1 participant).

Informants stressed that maintaining patient-centeredness in research study design is the overarching way to reduce participants’ burdens. All informants also generally agreed that participant comfort was paramount. Research teams should do “whatever is possible to maintain the comfort level of the participant, whatever that may look like” (HIV clinician #120), including “incentives, transportation, [and] going to their house” (HIV clinician #114). Further, participants should be recognized for their contribution and their time should not be taken for granted.

#### Considerations for next-of-kin/loved ones

We next asked informants to describe any considerations related to a participant’s next-of-kin (NOK) or loved ones. Early engagement of NOK/loved ones quickly solidified as a point of convergence:

*I think they [NOK/loved ones] would definitely need to be informed and fully involved with research study participation*. *They would need to understand*, *what is happening*, *what’s about to happen*, *especially if the intervention or whatever causes pain or hastens death … knowledge and preparedness of what it would mean for the participant to undertake participation in this study*.–HIV clinician #114

*[H]aving their loved ones engaged in the process of understanding what this means to the participant can help really improve the research experience across both the participant and their family and friends*.–Researcher #109

*All of the discussions around the intervention [and] the participation should be held jointly*. *The participant decides who their next of kin*, *loved ones are*. *They determine who the most important people are*. *When they’ve identified those people*, *they need to become part of the discussion*. *Just out of respect for the participant and also their loved ones*, *it’s just important that everyone be on the same page and communicating well*.–Researcher #113

Though there was widespread support for engaging NOK/loved ones, informants also universally recognized the participant’s right to decide whether and whom to include as part of the study:

*I go back to the agency of the participant*. *They get to decide*, *it’s their life…*. *I think you should always revert back to what the participant wants and finding a way to create a situation through their legal documents and then who has the medical power of attorney and all that to ensure that those wishes are honored*.–Community member #104

*[I]t’s not just involving the family*, *it’s making sure that if the patient doesn’t want the family involved*, *that the family is not involved*. *There’s both of those sides of the coin*.–FG-3 participant

*I think at the end of the day*, *it should be the participant’s choice*. *I mean*, *with the limitation again*, *that what we’re doing is safe*, *and does not induce pain…*. *I certainly don’t want to forbid or to impede their contribution to HIV science*, *particularly at this critical time*. *My priority really would be to listen to the participants*.–Researcher #117

The decision whether to involve NOK/loved ones was based partly on the emotional nature of this research (FG-3 participant) and on differing interpersonal dynamics:

*I think there’s a lot of family and partner dynamics that go on there that we would really need to explore because this is kind of really taking it to the next level*. *And I think this potential for a lot of discomfort*, *again*, *mostly on the part of the next of kin but to really be able to explore that deeply and the interactions and then how people feel about that*.–Community member #104

*The only thing I could see [that] might be problematic is the family relationships*. *They pose very interesting dynamics*. *Some families are more cohesive*, *some are very*. . . *they don’t talk to each other*, *basically*. *So that requires some very careful*, *empathetic*, *compassionate communication with all the family members involved*.–Researcher #108

The bioethicist diverged from other informants and stated, “we can’t justify putting the burden on the family if they aren’t comfortable with this decision [to undergo interventional HIV cure-related research at the EOL]” (#102).

We also queried informants as to whether NOK/loved ones should provide informed consent, not for the participant, but for themselves. We received responses both for and against such a measure. Informants provided several reasons in support of NOK/loved ones’ informed consent, namely to ensure that participants had discussed the research with their NOK/loved ones and that their NOK/loved ones agreed with the research and to prevent disagreements that may arise in the future. Those in opposition to having NOK/loved ones provide informed consent based their decision on the fact that a participant’s autonomy to participate in research should be respected. Their NOK/loved ones should only be required to provide informed consent if something was being asked of them, such as information related to their perceptions or experiences.

Research teams should involve NOK/loved ones of participants into the research process as early as possible and should communicate with them throughout the study, but only with participant consent. Research teams were also encouraged to pay attention to interpersonal dynamics, particularly since EOL research is emotionally charged.

### Considerations for specific HIV cure-related research strategies

#### Perceptions and safeguards around testing latency-reversing agents (LRAs) in the EOL translational research model

Informants generally considered LRAs as ethically permissible to test in the EOL translational model due to their established safety record in otherwise healthy volunteers (many LRAs are repurposed drugs from the oncology field), low risk profile, and the potential scientific knowledge to be generated:

*They are not so high*, *so super high risk*, *right*? *Compared to other interventions*. *So I will think that’s a good one to test in people at the end of life…*. *And one of the advantages of testing it with people at the end of life is that if we perform an autopsy*, *we might be able to see if the kick of the latency reversing agent might happen also in the tissue*.–Researcher #103

*I think that there are some very interesting latency reversing agents that would be very*, *scientifically if we were to be able to test them in people at the end of life*, *we would learn a lot*.–Researcher #109

Many informants did, however, express reservations regarding the potential efficacy of LRAs:

*Yes*, *with some reservations because in my experience they haven’t seemed to really work…*. *But that said if there were a new approach showing that it be of value then*, *then yes*, *absolutely*.–Community member #104

*Do we have any that work*? *… I’m game to try almost anything that might work*. *So sure*, *if we have one that works*. *Sure…*. *I don’t think either of those agents is ready for prime time*, *and the ones that we have are probably marginally effective*.–Researcher #119

Others described that the decision whether to tests LRAs in PWH at the EOL would depend on the specific agent and would depend on the possible side effects:

*Yeah*, *I would feel comfortable testing a latency reversing agent in someone at the end of life*. *There’s a caveat there*. *If it was a latency reversing agent that caused widespread T cell activation and made people feel really sick and miserable*, *that wouldn’t be my first choice*.–Researcher #113

*I would probably go with LRAs that have been tested previously in people who are on suppressive therapy and are all doing fine and the LRAs that did not reveal very serious side effects*.–Researcher #117

An HIV clinician (#111) agreed that testing LRAs in the EOL translational model was permissible, but noted that the benefits to be gained by testing at the EOL were unclear when compared to testing them in otherwise healthy PWH:

*I don’t necessarily know the benefit of doing them at the end of life…*. *I mean if it’s like the current study where you want to know does it affect … the reservoir in different tissues differently*, *then that makes sense*. *But that’s really more of a study of pathogenesis than an intervention efficacy test*.–HIV clinician #111

When asked which LRAs may be best suited to testing in the EOL translational model, our informants did not converge on any one answer. Instead, they pointed out that LRAs should not be tested alone because LRAs have already been tried by themselves and they did not prove efficacious at substantially reducing the HIV reservoir (HIV clinician #111 and Researcher #105). Informants also expressed the necessity of a well-reasoned scientific rationale for the selection of agents:

*Well*, *it all depends on the dosing and so on*. . . . *I think there’s excessive clinical trials*, *even in people who are well-controlled and otherwise healthy*, *because I think*. . . *a lot of the protocols I see are ones that I can’t conceive they’ll have any potential promise*.–Researcher #106

When pressed for specific agents to test, one researcher (#109) noted the precedent of testing histone deacetylase (HDAC) inhibitors in oncology. Another researcher (#105) expressed concern over testing HDAC inhibitors at the EOL because they were not perceived as very efficacious. Other informants remarked that second mitochondria-derived activator of caspases (SMAC) mimetics warranted additional *in vivo* data (Researcher #116) and should first be tested in otherwise healthy volunteers (Researcher #112) to determine their safety and efficacy before proceeding to testing them in participants at the EOL.

When questioned about safeguards necessitated by testing LRAs at the EOL, one informant (Researcher #103) stated that potential long-term effects of LRAs would be less of a concern in this population:

*Well*, *genotoxicity is something that we should not be worrying so much about the end of life*, *right*? *… Because usually carcinogenesis doesn’t happen so fast*. *And so at the end of life it’s less of a concern as it will be more a concern to me with young and otherwise healthy people with HIV*.–Researcher #103

Informants generally found LRAs acceptable for testing in the EOL translational model because of their established safety record, low risk profile, and the potential knowledge to be gained, yet expressed concerns over the potential lack of efficacy of LRAs. Many also noted that the selection of specific LRAs for testing at the EOL was highly dependent on the side effect profiles of the LRAs under consideration. Informants did not converge on any specific LRA that would be best suited to testing in the EOL translational model but rather expressed the necessity for a well-reasoned scientific rationale. They suggested careful monitoring and adequate pre-clinical safety data when testing LRAs in the EOL translational model.

#### Perceptions and safeguards around testing immune-based interventions in the EOL translational research model

Informants thought immune-based interventions would be ethically permissible to test in the EOL translational model mainly due to the significant knowledge to be generated:

*[Immune-based interventions] are good ones [to test] because [they are] relatively safe*, *[they have] been already tested in humans multiple times*. *And I think that one open question is tissue penetrability of this antibody… we could answer that with our translational research model*.–Researcher #103

Informants also believed the EOL translational model to be particularly suited to testing immune-based interventions because the potential for severe complications would be of lesser concern in an EOL population than in otherwise healthy PWH:

*I think [immune-based interventions are] a particularly good choice because they have the potential for severe complications through cytokine release syndrome … I think the immunological approaches to HIV… have the potential for complications that you’d first like to find out in a population like this*.–Researcher #118

One researcher (#110) advised caution, however, in testing immune-based interventions in PWH at the EOL because of the possible effects of comorbidities:

*[W]e don’t know how, especially depending on the clinical status of these donors, how they would respond because very often they will have comorbidities or other diseases, and so I’m a little bit, again and it’s my point of view, I would be very cautious about this modulation of the immune system*.

–Researcher #110

Others suggested that the data generated from such studies may be compromised:

*Sure*, *with the understanding that people who are at the end of life may be more immunologically compromised than otherwise healthy people so that the information may be compromised*.–Researcher #106

*I like the immune based approaches…[but] they may respond differently in people who are at the end of life versus persons who are not*.–HIV clinician #111

Some informants called for the comfort of the participant be given the utmost priority:

*If you had checkpoint inhibitors*, *that may cause an inflammatory complication that may increase the discomfort*. *I would have reservations about that*.–Researcher #113

An HIV clinician (#111) noted that considerations for testing immune-based interventions would depend on the specific intervention or study:

*[I]f this is with the intent to look at reservoirs in tissue*, *I think that that population really is the only group that you can acquire that from*. *So I think it would depend on the intent of the study*, *whether it was an intervention versus more like a basic science type study*.–HIV clinician #111

When asked which immune-based therapies would be best suited to test in the EOL translational model, broadly-neutralizing antibodies (bNAbs) were seen to be the best candidates due to their established safety profile:

*[M]y understanding is that those bNAbs are relatively safe*, *so I would be okay with the bNAbs that have been tested in people living with HIV in the past… [W]e now have*, *at least in monkeys*, *evidence that by combining bNAbs*, *you can really have a profound effect on … the viral rebound … but we don’t know much about tissue accessibility of these bNAbs*. *This is something that I think is critical*. *We actually never looked at whether those bNAbs can go into tissues in which we know HIV persist*, *such as the guts*, *such as the spleen*, *such as the lymph nodes*. *Using bNAbs in end-of-life participants is actually a great opportunity to answer these basic questions*.–Researcher #117

*Broadly neutralizing antibodies probably have the highest safety profile*. *There’s lots of evidence that you can pretty safely infuse antibodies*. *We use them for treatment all the time*.–Researcher #105

Researchers recognized chimeric antigen receptor (CAR) T-cells to be a riskier approach than bNAbs, but for both interventions EOL research could help assess penetration into deep tissues:

*I think that probably neutralizing antibodies or other agents that sort of weaponize the immune system*, *such as maybe even CAR T-cells or broadly neutralizing antibodies can give us a whole lot of information in the Last Gift model*. *In particular*, *you could think of adding both a latency reversal agent*, *so that you get the kick and then the CAR T-cells or the bNAbs to knock it out*. *Then the reason why they would [be] most interesting in the Last Gift model is that during the autopsy you could then go look for the broadly neutralizing antibody or the CAR T-cell*. *So you’d go see did it find its way to the brain*? *Did it find its way to the spleen where the HIV is hiding and all those different places*.–Researcher #109

One researcher (#107) noted that many of the monoloclonal antibody drugs (those ending in -*mab*) are immune checkpoint inhibitors (e.g. cemiplimab) meaning they may present greater safety issues and require more stringent safeguards due to the potential for unrestrained immune activation. As for the safeguards required for testing immune-based interventions, informants converged around increased monitoring, particularly for CAR-T cells and immune modulators ending in *-mab*:

*Well*, *neutralizing antibodies are relatively safe*, *but for CAR T-cells for example*, *there are cytokine storms*, *which are one of the side effects that can be lethal*. *And for example*, *usually when we do CAR T-cells infusion*, *we monitor people very closely for a fever and if there is any sign of cytokine storm*, *they get treated accordingly*. *You will want to check for fever*. *You will want to check for systemic inflammation*.–Researcher #103

*[H]ave a longer observation after the dose*, *maybe follow up with them for a few more days than you normally would just to kind of observe them and make sure that nothing’s happening*.–Researcher #107

*I think the only other safeguard that I would consider implementing is maybe even more frequent visits*, *or more frequent check-ins either by video visit*, *just to make sure that someone is feeling okay*. *Maybe a little bit more frequent lab values*, *based on the known side effects of the drug*.–HIV clinician #111

Overall, informants considered immune-based interventions as ethically permissible to test in the EOL translational model because the potential for severe toxicity would not be as much of a concern for people nearing the EOL. Some informants believed, however, that any immune data generated could be compromised by an EOL model. Informants also converged around bNAbs being the most suitable immune-based intervention to test at the EOL because of their established safety profile. Researchers described CAR-T cells as riskier than bNAbs but noted that both could assess the reservoir in deep tissues. Informants also recommended additional monitoring as an added safeguard when testing immune-based interventions at the EOL.

#### Perceptions and safeguards around testing cell and gene therapy approaches in the EOL translational research model

Informants expressed divergent views when queried about their perceptions of testing cell and gene therapy (CGT) approaches in the EOL translational model. Some reacted favorably to testing these approaches at the EOL because the long-term theoretical risks associated with CGT may not be as much of concern in an EOL population:

*I think that’s a good one to be tested at the end of life too*, *because there are some ethical concerns about carcinogenesis and also gene toxicity*. *And so since these are more long-term effects*, *I think that these are very good intervention[s] to be tested at the end of life*.–Researcher #103

One researcher (#105) noted that it may be difficult to adequately test CGT at the EOL because of unfavorable public perceptions around CGT and the need for a high number of trial participants to observe rare effects:

*Gene therapy … triggers a few people*. *So*, *I think it’s going to be tough*. *But if we think it’s working*, *let’s give it a try…*. *[Y]ou’re going to need a lot of people because maybe the side effects are rare … [and] they’re going to need to live long enough for you to sample and confirm that however many treatments you gave*, *reduce the reservoir by 10%*, *50%*, *90%*.–Researcher #105

One researcher (#117) stressed that it “might be too early to do that [test CGT approaches] in this population.” Other informants expressed unease about testing CGT approaches at the EOL because of the relative uncertainty associated with these approaches:

*I’m uneasy anyway to do any genetic manipulation in humans*, *so on top of it at the end of life*, *I’m a little bit uneasy about this*.–Researcher #110

*I think we really have no idea what would happen if we did gene therapy… I think there’s a lot of uncertainty*. *I would still be hesitant on that [because] … I have more doubt*.–Researcher #107

When asked the CGT approaches that they considered the best suited to test in the EOL translational model, our informants described various approaches. Among them, clustered regularly interspaced short palindromic repeats (CRISPR) was suggested as potentially having the best chance of success:

*[I]t’s pick your gene editing tool du jour which today*, *what’s always on the menu is CRISPR-Cas and then move it forward [because it] has some of the best chance[s]*.–Researcher #105

An HIV clinician (#111) suggested pairing zinc-finger nucelases with an effective delivery system that could be an alternative to CRISPR-Cas for targeting the latent reservoir.

As for safeguards necessitated for testing CGT approaches at the EOL, informants called for ensuring participants’ comfort at the EOL and extended close monitoring of participants, particularly for acute risks which may result in death. Likewise, the potential for “off-target” effects often associated with CGT concerned some informants:

*I think people are always a little worried with gene therapy*, *the potential off-target effects of gene therapy*, *where something else might be affected that’s not intended to be affected*.–HIV clinician #111

Whatever the case, informants recommended more careful deliberation and independent committee review before testing CGT approaches in the EOL translational model:

*I think there has to be a lot more deliberation*, *both by an independent review committee and much more careful*, *deliberate*, *informed consent*.–Researcher #106

Informants expressed divergent views around testing CGT interventions in the EOL translational model. Some responses were favorable due to the diminished concerns over theoretical risks associated with CGT. Others viewed CGT in a less favorable light because of the often unfavorable public perceptions of CGT, the limited pool of trial participants at the EOL, and the early-stage nature of CGT approaches. Informants suggested CRISPR gene editing and zinc finger nucleases as potential methodologies for testing in the EOL translational model. They also called for extended monitoring of participants, particularly for acute risks which may prove fatal.

#### Perceptions and safeguards around testing stem cell therapies in the EOL translational research model

When asked about conducting stem cell transplants in the EOL translational model, informants strongly diverged in their opinions. Most informants did not believe it ethically permissible to perform stem cell transplants at the EOL because of their high risk profile and the significant discomfort they would cause to participants:

*[T]hat’s probably where I draw my line*. *I don’t think that’s a good idea because there is too much acute toxicity with stem cell transplant*. *And I do think that there is a very high likelihood that people that are already at the end of life might die from a stem cell transplant*.–Researcher #103

*I’m not signing up for a stem cell [transplant]*. *The toxicity of stem cell transplants is so high*. *You’re taking people at the end of life*. *I’m not sure they’ll live through the stem cell transplant*. *So*, *there’s a whole lot of toxicity to get through the transplant to figure out*. . . *Again*, *when you go to risk benefit*, *I think this is one where the toxicity of the intervention is so high that people have to live through the stem cell transplant to see if there’s a benefit of reconstituted bone marrow*. *I can’t see doing that*, *particularly when*, *again*, *the preliminary data is terrible*. *We’ve got an n of two I think right now of stem cell transplants that have survived*.–Researcher #119

Other informants who were opposed to testing stem cell transplants remarked that the scientific knowledge to be gained may be great except that we may not be able to observe the full effects of a stem cell transplant in an EOL population and that the inherent risks remained too high:

*Here’s an area where you might learn a whole lot about what it takes to get something truly reproducible to eliminate a reservoir*. *But to do that*, *that gets to be a point where you know some of the interventions*. *They’re probably going to be very*, *very close to lethal*.–Researcher #116

*The problem with this type of intervention is that the transplant takes time to really disseminate throughout the body and persists, so if the patient is about to die within the next few weeks, I would say that would probably not give you enough time to really appreciate the effect of the transplants*.

–Researcher #117

Still others remarked that the cost prohibitiveness and high risk profile (HIV clinician #111) of such a procedure would significantly hamper the scalability of stem cell transplants and, thus, render them non-viable as a global HIV cure strategy:

*Not really, because I really don’t think that’s a viable mechanism for going forward with cure for HIV… [W]e’re not going to do this to 35 million people to try to cure their HIV, that’s not reasonable*.

–Researcher #105

A minority of informants (Researchers #101, 107, and 108), however, stated that testing this modality would be permissible. A researcher (#105) who was opposed to testing stem cell transplants did add an exception in the case of when the transplant was already clinically indicated (e.g., a patient with concomitant HIV and malignant cancer).

Most informants were adamantly opposed to testing stem cell transplants in the EOL translational model, except where already indicated for cancer, because of the high risk profile, significant discomfort, and high cost of such an intervention. They also noted that realization of effects may be limited at the EOL and that such a strategy was not likely to be globally scalable. Only a small minority of informants stated that this intervention may be ethically permissible at the EOL.

#### Perceptions and safeguards around testing combination approaches in the EOL translational research model

Informants converged on a favorable view of testing combination approaches in the EOL translational model, recognizing that such an approach was “probably our best chance” (Researcher #105) for finding a cure for HIV:

*[A]s a matter of fact*, *it’s my opinion that those are the only kinds of interventions that are likely to be effective*, *analogous to the situation with use of combination [ART] therapy*.–Researcher #101

*That’s the kind of thing that would make sense to me*, *but I think*, *again*, *you’re looking for a big return on investment in a short time period*. *These kinds of studies need to be structured so that you’re looking for a big return in a short period of time*.–Researcher #119

Informants also noted that the ethicality of testing combinatorial regimens depends on the unique combinations being tested (Researcher #112) and should not significantly affect the participant’s QOL (Researcher #110).

One researcher (#119) preferred to test monotherapies in the EOL model before proceeding to combination therapies:

*I’d probably start with individual therapy first. If we’re going to use CAR T-Cells, we ought to figure out how that works. A works, B works, put A and B together before I’d start doing combination therapies*.

–Researcher #119

Yet another researcher preferred to test combinations in otherwise healthy PWH before testing them in terminally-ill PWH:

*Combinations. Why not, but why don’t we do that first in people that are not about to die? That’s always the same thing. I think it would be safe and actually probably a pretty good idea to try to combine these different drugs in people living with HIV. Do we need to do that right now in people at the end of life?*

–Researcher #117

When asked to describe the best suited combination approach to test in the EOL translational model, informants pointed to potentially one or more LRAs combined with a clearance mechanism (Researchers #109 and 116), such as bNAbs or CAR-T cells:

*I think the kick and kill approach makes a lot of sense to me*, *but again we don’t seem to be able to kick them all out*. *So if we can get something that can really target only HIV infected cells*, *and kick those without mounting a huge immune response with all of the T-cells*, *and then just let those awakened cells be identified and maybe then destroyed by CD8 T-cells that have been enhanced by immunotherapy*. *That would be the ideal*, *but we don’t have anything quite like that*.–HIV clinician #111

*Like a latency reversing agent together with a neutralizing antibody*. *Latency reversing agent with CAR T-cells*. *Right*. *So I will think the best combination that you can see is something that kicks together with something that kills*.–Researcher #103

One researcher (#110) was reluctant to include an immune-based modality in a combination because of the high potential for side effects. Informants also pointed out that, when testing combinatorial approaches, researchers should resist the urge to combine multiple agents with no scientific rationale for additive or synergistic effects (Researcher #106) and should carefully monitor participants for side effects (Researcher #103).

Overall, informants expressed that combination approaches are most likely to lead to an HIV cure and were in favor of testing them in the EOL translational model. Some informants urged caution in proceeding with combinations and recommended testing monotherapies first. Latency reversing agents in combination with a clearance mechanism was considered the best-suited combination approach for testing in the EOL population, so long as there was a strong scientific rationale for combining these agents.

#### Perceptions and safeguards around testing novel approaches in the EOL translational research model

Informants reported two novel approaches for an HIV cure they believed may be ethically permissible to test in the EOL translational model: therapeutic interfering particles (TIPs) and adeno-associated virus (AAV) as a vector for CGT approaches.

TIPs are defective HIV particles that theortetically work in competition with HIV [28]. TIPs lie dormant within the body and only activate when HIV begins to replicate [28]. TIPs could prevent HIV replication by consuming all of the building blocks on which HIV relies in order to replicate (Researchers #109 and 112). TIPs could also transmit from person to person, but only among PWH (Researchers #109 and 112). Testing these at the EOL could provide significant scientific knowledge around efficacy and where TIPs concentrate in the body:

*[P]robably the first place it needs to be tested in humans anyway*, *would be in sort of this Last Gift project [EOL population]*. *One*, *we would be able to see if it actually worked*. *Then two*, *we would actually be able to see where those interfering particles might go*.–Researcher #109

One researcher (#116) cautioned restraint because of the high level of uncertainty associated with such novel interventions:

*I don’t know whether that one is ready for prime time yet and I would think that end of life is prime time*. *Is there enough animal data*, *are we satisfied that there’s enough in vitro and animal data to get that answer and I’m still not ready for that one*. *So*, *I don’t think I could do that*.–Researcher #116

Other researchers (#109 and 112) acknowledged the uncertainty (e.g., inflammatory response and escape mutations) and ultimately supported testing this intervention at the EOL:

*[These] interfering particles … [have] been used in infectious diseases research*, *but mostly around vector-borne diseases*. *[W]e don’t know does HIV have a secret mechanism to escape from [TIPs]*? *We thought that when the first HIV drugs were developed*, *we didn’t think about resistance too much*. *When HIV was a wily adversary*, *so it found a way to get around it*. *We don’t know if it would also find a way to get around one of these interfering particles… But the only way that we would ever know that is to test it*.–Researcher #109

A concern was expressed over TIPs transmission between PWH. One researcher (#112) recognized this as a valid concern for some, but also noted that this concern was likely assuaged by the live attenuated vaccine debate:

*[T]he very first ethical challenge that almost everyone hones in on is a transmission*. *You’re introducing a therapy that transmits between people*, *and for most people*, *it’s an immediate showstopper… Epidemiologists are excited by it because it’s a new way of trying to control population level infectious disease*. *And there is precedent for therapies that can transmit in this way and the best precedent are live attenuated vaccines*. *So*, *all of the safety issues and transmission issues … in essence have already been dealt with in the live attenuated vaccine debate*, *about whether you can release live attenuated vaccines*, *which we know will transmit in a limited fashion between individuals*.–Researcher #112

Potential concerns may exist over the theoretical risk of “insertional mutagenesis, where you’ll cause cancer because you’re introducing an HIV like virus which will integrate in the genome” (Researcher #112), although this has not been shown to be a problem with lentiviral vectors (Researcher #112).

As with any intervention, robust pre-clinical data would be required (Researcher #116). Participants in human trials would also need to be closely monitored (Researcher #112). In case of any untoward effects with TIPs, re-starting ART would act as the off-switch:

*Since they are dependent on the virus*, *the off switch are the 40 or more approved antiretroviral drugs that exist*. *So*, *if the patient goes on antiretroviral therapy*, *that will shut off the virus*, *which will inherently shut off the interfering particle*, *which is the same chassis as the virus*. *And dependent on the virus*.–Researcher #112

The other novel approach that emerged in our study, AAV vectors for CGT approaches, was briefly mentioned by two informants (Researchers #109 and 117) as a possible candidate for testing at the EOL. One researcher noted that testing this approach in an EOL population would not answer questions regarding longevity of AAVs but could inform where the AAVs concentrate in deep tissue compartments. Another researcher expressed concern over testing AAVs at the EOL because these interventions remain in the early-stage.

Informants expressed the ethical permissibility of testing novel HIV cure research approaches in the EOL translational model, such as TIPs and AAVs. Informants acknowledged the uncertainity and early-phase nature of both of these novel interventions, noting the need for additional robust pre-clinical data.

#### Perceptions and safeguards around analytical treatment interruptions in the EOL translational research model

Generally, informants perceived the use of ATIs in the EOL translational research model favorably because ATIs are currectly “the only way to determine if a cure intervention has been effective” (Researcher #101). Further, ATIs have a high safety record in otherwise healthy PWH, and many PWH near the EOL already stop ART on their own:

*Sure. Most people do them during life, so I don’t see any reason not to do them at the end of life*.

–Community member #104

*[A] lot of people already do it [treatment interruption] by themselves because a lot of people don’t really want to be on antiretroviral therapy at the end of life*.–Researcher #103

*I think this is kind of an ideal patient population*, *because a lot of my concerns in other patient populations about re-seeding the reservoir and bad things that can happen I don’t have in this patient population*.–Researcher #119

*Analytical treatment interruptions can be done safely even in non-end-of-life clinical research studies and have been done so for years*. *In the proper context*, *in a rigorous clinical trial with a robust safety precautions*, *yeah*, *they’re safe enough*.–HIV clinician #114

Some informants expressed concerns over the use of ATIs in terminally-ill PWH. One researcher (#107) was hesitant about using ATIs in an EOL population because, “this is already a vulnerable population … [that is at] higher risk of having negative symptoms” which would increase “burden on the staff … to protect them from … an acute viral illness.” A researcher (#117) noted that the scientific knowledge to be gained may be limited unless ART was stopped very near the EOL:

*[When] you stop ART*, *virus comes back [and] disseminates pretty much everywhere*. *Then eventually these people die*, *then you can collect the tissues*, *but there is no way you can identify the source of rebound because it’s too late*, *the virus already spread pretty much everywhere*. *I think we can actually learn more by maintaining antiretroviral therapy until the end*, *and then look carefully at every single tissue*, *and use very sophisticated assays to determine where the live virus is… The only exception… is if you stop ART really close to death*, *when the virus just comes back to replication*, *[then] maybe you have the ability to actually identify the tissues in which this resurgence happens*.–Researcher #117

According to informants, the use of ATIs in EOL HIV cure trials conducted necessitate additional safeguards. Foremost among these is ensuring participants are well informed of the associated risks and the manifestation of viral rebound:

[*P]eople need to understand that they could have a reaction if they were to have a rebound and then what those reactions look like*, *fevers*, *chills*, *sore throat*, *acute retroviral syndrome*.–Researcher #109

Some informants (Researchers #105, 106, and 112) also recommended close monitoring procedures be followed when conducting an ATI containing trial. One HIV clinician (#115) noted that a data safety monitoring board (DSMB) may be appropriate:

*This is when the role of a DSMB would be interesting to me*, *because I don’t think it would look like a regular DSMB because obviously*, *there could potentially be more things that are tolerable even though they look disproportionate*. *But I wonder if you can have some sort of modified DSMB*.–HIV clinician #115

Another HIV clinician (#111) mentioned that the care provider should also be brought on-board if an ATI were to be performed. Other informants cautioned that participants could risk transmission of HIV to sexual partners but noted this is unlikely at the EOL. In any case, one HIV clinician (#111) recommended counseling around partner protection measures to anyone who would do an ATI.

Overall, informants shared a favorable view of ATIs used in HIV cure research at the EOL because they are currently the only method available for quantifying an HIV cure intervention’s effect and have an established safety profile in otherwise healthy PWH. Some informants expressed concern over the use of ATIs in an EOL population because this population may be seen as vulnerable, may be more susceptible to adverse events, and the scientific knowledge to be gained may be limited. Our informants expressed the need to inform participants in ATI trials about the associated risks and manifestation of viral rebound, as well as the need for close monitoring and counseling about the use of condoms to prevent transmission of the virus to sexual partners. They also noted that participants always had the option of restarting ART.

### Additional considerations

#### Ascertainment of death as a serious adverse event

Death is considered a serious adverse event (SAE) in clinical research. In EOL translational research where death is an inevitability, the ascertainment of death as an SAE may be complicated (Bioethicist #102). We asked informants how to deal with this “tricky” issue (Bioethicist #102). Most informants believed that death would be an expected adverse event and, thus, converged on the view that the cause of death (i.e., the terminal illness versus the intervention) should be determinative:

*The principal way would be … is death … a[n] unexpected adverse event in this particular situation*? *It could occur at any time as part of the natural history of the underlying illness*, *or it could be precipitated by whatever treatment intervention is being offered to the participant*.–Researcher #101

*I don’t consider all death SAE*. *I think to a large extent it depends on how they died*. *If something precipitates their death that was unexpected or something like that*, *then sure*. *But I don’t think all death is an SAE*, *even if they’re on a research study*.–Researcher #119

Informants also generally agreed that a determination of the cause of death should be made by the research team. The determination of the cause of death should also be transparent and reviewed by an independent body:

*It seems to me that there’s that teasing out*, *what was the cause would be difficult*. *And so my go to answer when you aren’t sure is to declare*, *to disclose*, *to ask*, *to make it transparent*.–Bioethicist #102

*I think you always report SAEs, even if they are unlikely to be a direct cause of the intervention itself. So they have a terminal illness, and they died of that terminal illness. You would still report an SAE, but I think any reasonable person on the IRB would understand that they died of their [underlying] illness*.

–Researcher #113

A researcher stressed the need for transparency around the reporting of all deaths (#103). One HIV clinician noted the potential use that such information about death could provide in EOL research:

*Ultimately*, *these results will be translated into non end-of-life populations; having a marker of death*, *timing of death*, *frequency of death in these studies*, *might serve some information for future research studies*.–HIV clinician #114

When ascertaining whether a death in an EOL HIV cure trial should be considered an SAE, informants were of the general opinion that the cause of death would be determinative. In most cases, death would be an expected SAE. They also stressed the need for transparent reporting procedures and independent review of the cause of death.

#### Participants with concomitant conditions at the EOL

There was a divergence of opinions as to whether interventions should be tested in individuals with concomitant conditions at the EOL. Community members focused on the participant experience of helping inform scientific knowledge on more than one disease, such as cancer and HIV:

*Any way we can leverage the research to accomplish more*, *I think it would be a good thing*. *It’s more complicated*, *I think on the research side*, *but I don’t see any downside for participants*. *I think they would be thrilled to realize they could help in more ways than one*.*… You know*, *we’ve had a number of ALS patients*, *so I’m sure they would be thrilled if they thought that*, *not only could they help with the HIV cure effort*, *but they could help with research towards finding a cure towards the ALS*.–Community member #104

Likewise, some HIV clinicians and researchers noted that research was already being done in PWH at the EOL with concomitant conditions:

*Timothy Ray Brown [e*.*g*., *the “Berlin Patient”–the first person cured of HIV] had an aggressive cancer that would have killed him but had a unique opportunity to get the delta 32 bone marrow transplant that would ultimately result in a functional cure and a treatment of the underlying disease*.–Researcher #101

*If you have [a] cancer study, and you have somebody with HIV, and you think that you can make a difference in latency, then you should have double outcomes for sure. I think that totally makes sense*.

–HIV clinician #111

Other HIV clinicians and researchers, however, cautioned restraint because of the confounding effects of the data, limited resources, and disease-specific sources of funding:

*Oh*, *let’s go slow*. *Maybe*, *but I think it gets complicated*, *because as with everything*, *there are confounding issues*. *End of life research is already hard because there are so many things we cannot control*. *We cannot control for all of the other things that are going on in the person’s life… [O]ne of the biggest challenges with end of life research is the confounding*, *is the fact that we’re treating one disease but we’re not randomizing participants*. *We’re not bringing people in controlling for age*, *race*, *ethnicity*, *obesity*, *tobacco*, *years on HIV therapy*, *type of HIV therapy*, *size of reservoir*. *None of that*.*… I think it is already a science that needs a lot of statistical help*, *and I think to add another disease condition to it will make it a thousand times harder*. *It doesn’t mean you can’t do it*. *It just means let’s make things as easy as possible so that the results are interpretable as much as possible*.–Researcher #119

*If the question [and the funding] is related to*, *let’s say*, *HIV*, *then you’d want to be focusing on that with the limited resource that you have*.–Researcher #118

With regard to testing interventions in people with concomitant conditions at the EOL, informants expressed divergent opinions. On the one hand, this research has already been done on people with concomitant conditions and participants could feel a greater sense of self-fulfillment by contributing to research on more than one disease. On the other hand, the confounding effects of multiple comorbidities, non-randomization of participants, and limited resources support the exercise of caution.

## Discussion

Our qualitative study probed key informant perspectives on the ethical and practical considerations of conducting interventional HIV cure-related research at the EOL. As previously stated, observational HIV cure-related studies at the EOL are already underway [3]; testing interventions in the EOL population would be the next logical scientific progression modeled on the field of oncology [29–31]. Our results demonstrate generalized yet conditional support among all key informant groups regarding the ethicality of this research. Conditions included a clearly delineated respect for participant contribution and autonomy in engaging in interventional research at the EOL, participant understanding and comprehension of the inherent risks associated with the specific intervention(s) to be tested, and broad community support for testing the proposed intervention(s).

Interventional HIV cure-related research at the EOL holds the promises of both societal and personal benefits. On the societal side, the potential scientific knowledge to be gained would be vast. This knowledge could result in strides towards finding an HIV cure and the effects of interventions in deep body compartments. The personal benefits would not be easily quantifiable, but would nevertheless have notable impacts on participants [32]. Attitudes are changing to recognize people nearing the EOL as autonomous individuals rather than members of a vulnerable population [33, 34]. As evidenced with the Last Gift study, the participation of terminally-ill PWH in this research confers a deep sense of meaning and purpose [35–37]. Scientific altruism, or the desire to help others in the future, is a motivating factor in HIV cure-related research at the EOL and has long been a hallmark of the HIV/AIDS community [3, 38, 39].

Despite the potential scientific benefits of interventional HIV cure-related research, this research may have many potential risks as well. Among them would be the potential for increased suffering, decreased QOL, and accelerated death of study participants. Although these risks may seem significant to some [36], the ultimate decision of what would constitute too much or unacceptable risk should remain with the study participants, particularly since having control and autonomy often becomes increasingly important at the EOL [40]. There also exists the potential for scientific risks of generating data that are non-generalizable given small sample sizes and the fact that interventions may not have the time to manifest their long-term effects.

Recognizing that any research at the EOL is sensitive and emotionally charged [35, 41], testing interventions which offer no hope of direct clinical benefit to participants at the EOL may be seen by some as morally wrong. Further, any adverse effect, whether expected or otherwise, may potentially cause a public backlash. To prevent such a situation from occurring, robust community engagement and education will be necessary [40, 42] from inception to completion of any interventional HIV cure-related study.

To ensure acceptable benefit-risk profiles for interventional HIV cure-related research at the EOL, one must assume that unquantifiable personal benefits are weighted based on the participant’s autonomy and not, as some researchers have suggested, simply *de minimis* [43]. Study teams, therefore, should focus their attention on minimizing risks such as undue pain and suffering, as well as reducing the likelihood of unintended effects. Researchers should develop protocols that only test well-vetted interventions that are supported by robust pre-clinical data. Participants’ clear understanding and comprehension of the possible risks associated with proposed intervention(s) are also integral to ensuring acceptable benefit-risk parameters. This necessitates a robust informed consent process [35, 40] that includes process consent, a procedure that allows for consent to be renegotiated throughout the study cycle [36]. Our data also show a consistent call for multi-disciplinary research teams that include biomedical research, bioethicists, socio-behavioral scientists, and community members working collaboratively to ensure such research remains ethical.

Further, our findings reveal that the comfort of study participants should be the utmost priority of research teams conducting interventional HIV cure-related research at the EOL. Participants’ time should under no circumstances be taken for granted. A patient/participant-centered approach that minimizes participant burdens [40] is also essential to community and patient/participant acceptance of HIV cure-related research at the EOL. Protocols should be developed to remain flexible and/or adaptable to account for individual participants’ circumstances [35], even if this results in a greater burden for the research staff. Whenever possible, research staff should travel to the participants’ location for study visits [36, 40] and, when not possible, staff should maximize the scientific utility of in-person study visits.

One ethical tension presented with interventional HIV cure-related research at the EOL is the need to balance NOK/loved ones’ desire for information/participation with the paramount autonomy of the study participant [44, 45]. General consensus exists in the research literature that EOL care and, by extension, research should focus on the NOK/loved ones, as well as the participant themselves [41, 42, 46]. Our findings show that NOK/loved ones should be involved early in the research process if and only if the participant consents. Should such consent be obtained, research teams should communicate regularly with NOK/loved ones throughout the study. Since EOL research is such an emotionally charged topic [41] and HIV remains a stigmatized disease, the interpersonal dynamics of each situation must be taken into consideration before involving NOK/loved ones.

Turning to specific HIV cure-related research strategies, initial interventions tested at the EOL would mostly provide foundational information on such strategies and not likely offer a chance of curing HIV. Informants provided thoughtful considerations and safeguards to ensure the ethical permissibility of implementing HIV cure-related interventions at the EOL. Our data clearly demonstrate that the ethicality of testing specific HIV cure research strategies at the EOL depends on each intervention and must be based on a strong scientific rationale and robust pre-clinical data (and clinical data in otherwise healthy volunteers).

While LRAs may be ethically acceptable in PWH at the EOL, their lack of efficacy in otherwise health volunteers reduced enthusiasm for this approach. Immune-based strategies possess the potential for great scientific gain, although any data generated may be skewed by compromised immune systems in an EOL population. Likewise, CGT strategies should be based upon ample pre-clinical data to support testing them at the EOL, specifically because of generalized public hesitancy over CGT approaches [47]. Combination strategies were viewed by some informants as having a greater chance of providing a cure for HIV over monotherapies. Before testing combination approaches, however, monotherapies should be tested so that a scientific rationale for combining them can be ascertained. Novel approaches, such as TIPs or AAVs, will require additional pre-clinical data before being tested in humans, especially those nearing the EOL. Finally, except where already clinically indicated [48], stem cell transplants posed too high a risk and may be too cost-prohibitive to even be considered for testing in the EOL translational model.

Informants stated that ATIs are currently ethically acceptable for use in participants at the EOL because they are the only means so far of measuring an intervention’s effect. We previously argued that PWH at the EOL should not explicitly be asked to interrupt ART in purely observational studies, but may elect to do so of their own accord [3]. In interventional studies conducted at the EOL, however, we took the position that ATIs were permissible as part of a protocol [3]. This study confirms our initial position: informants noted that ATIs should be used when necessary to the protocol and after extensive consent in which the participant is informed about the inherent risks and manifestation of viral rebound, as well as the potential for transmission of HIV to sexual partners.

Additionally, the resultant findings from questioning about the ascertainment of death as an SAE pointed to the cause of death as being determinative in HIV cure-related research at the EOL. Since death would be expected when working with terminally ill volunteers, all deaths may not necessarily be considered adverse events. Informants called for guidelines for reporting procedures for deaths, as well as an independent review of the cause of death.

A divergence of opinions resulted regarding the viability of testing HIV cure-related interventions in people with concomitant conditions at the EOL. On the one hand, results showed this research has already been done in patients with HIV and cancer (e.g., the Berlin and London patients [49, 50]) and that participants would likely feel a greater sense of self-fulfillment in contributing to research on more than one condition. On the other hand, results also revealed the potential for confounding findings and the difficulty of implementing such research given disease-specific funding streams.

The summary of ethical and practical considerations for interventional HIV cure-related research at the EOL derived from our empirical research study can be found in Table 4. This list may not be exhaustive.

**Table 4:** Summary of ethical and practical considerations for interventional HIV cure-related research at the EOL.

**Testing Interventions in the EOL Translational Research Model**
**Ensuring Ethical Permissibility of Interventional HIV Cure-Related Research at the EOL**
• Research teams have an obligation to design HIV cure-related studies at the EOL that ensures participants’ understanding of the risks involved for a particular intervention. Protocols should be vetted at multiple levels, and there should be robust community input in designing the research protocol.
• Participant’s autonomy in decision-making should remain paramount.
**Maximizing Benefits of Testing Interventions at the EOL**
• Research teams should strive to maximize the benefits from interventional HIV cure-related studies at the EOL, both societal benefits such as knowledge generation and advancement towards an HIV cure, as well as the participants’ psychosocial benefits such as personal fulfillment and satisfaction.
**Minimizing Risks of Testing Interventions at the EOL**
• Research teams have an ethical obligation to minimize participant risk of increased suffering, decreased QOL, and accelerated death when testing interventions at the EOL.
• Research teams should ensure robust, generalizable data and remain cognizant of the public’s perception of this research.
**Ensuring Acceptable Benefit/Risk Profiles for Testing Interventions at the EOL**
• The EOL context may alter the benefit-risk assessments; still, there should be upper limits on acceptable risks. Research teams should strive to find interventions with the greatest potential for scientific benefit with the relatively lowest risk of participant harm.
• There should be adequate preclinical data before testing interventions in humans at the EOL. Studies should begin with the most conservative dosage to ensure that the intervention does not cause pain or have unnecessary side effects.
• Research teams should be multi-disciplinary in nature and include biomedical researchers, community members, care providers, bioethicists, and socio-behavioral scientists.
• The informed consent process should be deliberative and institute “process consent,” an ongoing consent process throughout the study. Participants cognition should be tested multiple times throughout the trial to ensure they truly understand and comprehend the risks and benefits of their participation in the trial.
**Understanding Perceptions of Unacceptable Risk**
• To ensure interventional research at the EOL continues to remain ethically permissible and socially acceptable, researchers will need to understand stakeholders’ perspectives of what is acceptable versus unacceptable risks and burdens. Research into empirical research ethics and the socio-behavioral sciences will be necessary to understand evolving perspectives about this type of research.
**Minimizing Participant Burdens at the EOL**
• Research teams should reduce burdens to study participants through adaptive, participant-centered protocol designs that prevent undue suffering at the EOL (e.g., travelling to the participant).
**Integrating Considerations for NOK/Loved Ones**
• Next-of-kin/loved ones should be brought into the research process as early as possible. There should be open communication with them throughout the entire process, but only with participant.
• Because this research deals with the emotionally charged topic of EOL, research teams should pay particular attention to partner/family dynamics.
**Considerations for Specific HIV Cure-Related Research Strategies**
• interventions in PWH at the EOL is highly dependent on specific interventions and must be based on a strong scientific rationale supported by robust pre-clinical data and/or clinical data in otherwise healthy volunteers.
• Frequent and extensive monitoring of participants is necessitated to ensure the safety of participants with HIV at the EOL. Monitoring should not be so intrusive that it adds burdens for participants or jeopardizes participants’ comfort.
• LRAs have shown limited efficacy to date and may present clinical risks. They should preferably be tested in combination with clearance strategies.
• Research teams must recognize that any data generated by testing immune-based interventions may be compromised by testing in an EOL population.
• Due to the early-phase nature of CGT research and prevailing public perceptions of CGT, robust pre-clinical data must first be ascertained that demonstrate a solid scientific rationale before testing CGT at the EOL.
• Except where already clinically indicated (e.g., cancer), research teams should not test stem cell transplants at the EOL because of the significant risks posed.
• Combination therapies are likely to increase the chance of finding regimens that can keep HIV suppressed without ART. However, there should be a strong scientific rationale for using each strategy in combination.
• There should be robust pre-clinical data and/or clinical data in otherwise healthy volunteers before testing novel approaches in the EOL translational model, such as AAVs. TIPs should probably be tested in the EOL population first since TIPs can transmit between people.
• Research teams may use ATIs in EOL research after a robust informed consent process. Research teams should educate participants in ATI trials about associated risks and manifestation of viral rebound, as well about the potential forward transmission of HIV.
**Additional Considerations for Interventional Research at the EOL**
**Ascertaining Death as a Serious Adverse Event**
• The cause of death in the EOL translational model, as determined by the research teams and reviewed by an independent body, should determine whether death should be classified as an SAE.
**Conducting Research with Participants with Concomitant Conditions at the EOL**
• There may be scientific benefits to studying two concomitant conditions (e.g., HIV and cancer); however, this may also increase the likelihood of confounding factors. Disease-specific funding streams may preclude such research.

### Limitations

Results from our qualitative study must be interpreted in light of their limitations. First, saturation (the point at which no new themes are observed in the data [25]) may not have been reached after 20 interviews and 3 focus groups. Our sample was constrained to a relatively small number of informants due to time and funding constraints. Further, our recruitment by stakeholder group was skewed, and there was an imbalance with respect to the informants who were available to participate in our study during the COVID-19 pandemic. Second, due to our purposive sampling technique, our informants were in general supportive of testing interventions in the EOL translational model. We acknowledge that dissenting opinions abound and that further inquiry is necessary to accurately capture these views, particularly in culturally diverse populations. Without such input, the generaliziability of the current work is limited given that most informants were Caucasian. Perceptions of this research may be quite different within communities of color. More research will also be needed to satisfy the ethical considerations of justice and equity with respect to access and opportunity to participate in HIV cure-related research. Third, our considerations are likely skewed to resource-rich contexts. Our considerations further represent the views of a well-informed, older, and predominantly white population. Thus, more research is also needed to understand the opinions of diverse populations on interventional HIV cure-related research at the EOL. Finally, COVID-19 has undoubtedly skewed our data by limiting the participation of HIV clinicians and researchers at the time of data collection. Our study was formative in nature; as such, we focused on *broad* categories of HIV cure-related research interventions that could potentially be tested in PWH at the EOL. More research will be necessary to determine practical and ethical considerations for testing *specific* interventions in the EOL translational research model (e.g., small-molecule repurposed agents such as JAK 1/2 inhibitors [51], CGT approaches such as CRISPR-Cas9 [52–54], long-acting slow effective reslease ART [55, 56], etc.). Ethical research at the EOL will require robust and sustained engagement of diverse communities, patients, clinicians, and NOK/loved ones. We should never abandon the question of what makes such research ethical.

## Conclusions

To our knowledge, this is the first study to generate ethical and practical considerations for implementing interventional HIV cure-related research at the EOL. We hope our findings will serve as a foundation for future research, dialogue, and community-engaged research on this topic. Inevitably, science will continue to progress towards an HIV cure. A strategic next step will likely be to test promising interventions in PWH at the EOL, to the extent these practices are deemed ethically and socially acceptable. Future research will be necessary to examine the ethicality of testing *specific* interventions in PWH at the EOL. The field may need to develop a robust rubric to guide the ethics review of these protocols that details acceptable interventions and critical safeguards, as well as safety and efficacy parameters. The ethical and practical dilemmas of such research must be considered now. PWH at the EOL should be allowed to exercise their autonomy and meaningfully contribute to the search towards an HIV cure. They should not, however, be asked to participate in high risk research with little or no likelihood of significant gain in scientific knowledge. We must remain good stewards of their extremely altruistic gifts by maximizing the impact and social value of this research.

## Supporting information

S1 Table Ethical and practical considerations for interventional HIV cure-related research at the EOL. (DOCX)

## Abbreviations

AAV: adeno-associated virus
AIDS: acquired immunodeficiency syndrome
ART: antiretroviral therapy
ATI: analytic treatment interruption
AVRC: AntiViral Research Center
bNAb: broadly neutralizing antibody
CAB: community advisory board
CAR: chimeric antigen receptor
COREQ: consolidated criteria for reporting qualitative research
CGT: cell and gene therapy
CRISPR: clusters of regularly interspaced short palindromic repeats
DSMB: Data and Safety Monitoring Board
EOL: end-of-life
HARP-PS: HIV + Aging Research Project–Palm Springs
HDAC: histone deacetylase Inhibitors
HIV: human immunodeficiency virus
HIPAA: Health Insurance Portability and Accountability Act
IRB: Institutional Review Board
LRA: latency reversing agent
NOK: next-of-kin
PWH: people with HIV
QOL: quality of life
SAE: serious adverse event
SD: standard deviation
SMAC: second mitochondria-derived activator of caspases
TIPs: therapeutic interfering particles
UCSD: University of Southern California–San Diego
UNC-CH: University of North Carolina–Chapel Hill
