# Supplementary material for: Ethical and practical considerations for interventional HIV cure-related research at the end-of-life: A qualitative study with key stakeholders in the United States
Source: PLoS One. 2021 Jul 16;16(7):e0254148. doi: 10.1371/journal.pone.0254148 (PMC8284787; doi:10.1371/journal.pone.0254148)
Supplement: S1 Table — (DOCX) [file pone.0254148.s001.docx]

**S1 Table. Ethical and practical considerations for interventional HIV cure-related research at the EOL.**

| **Themes and Sub-Themes** | **Participant Number** | **Informant Type** | **Exemplar Quotes** |
| --- | --- | --- | --- |
| ***Testing Interventions in EOL Translational Research Model*** | | | |
| **Ensuring Ethical Permissibility of Interventional HIV Cure-Related Research at the EOL** | | | |
| Ethical permissibility of testing of higher-risk interventions | 107 | Researcher | *I think maybe especially with some of these more extreme medications, not even extreme but just some of the ones that have a higher risk profile like they're using it for cancer therapy and now we're trying them in HIV research. So maybe start with a population that is less impacted like long-term.* |
|  | 116 | Researcher | *Many of the things that we consider might have a very long term consequence, but we don't have to think about in terms of a risk. If we eliminate that risk and it tips the balance to benefit the wider community, because this is all going to be wired at communities. I think for those reasons it is possible.* |
| Recognizing participant autonomy | FG-1 | Participant | *[I]t should be something that is up to the participant… And they should really be well-aware of some of the side effects that these things can cause.* |
|  | 104 | Community member | *I keep saying this but recognizing their agency and making these decisions participating as partners in the research…it's really a choice and a partnership between the participant and the research team.* |
|  | 115 | HIV clinician | *There's no question end-of-life care research with interventions will take a very special and unique person.* |
| Recognizing ethicality depends on specific interventions | 102 | Bioethicist | *The question is, how much suffering is acceptable? I mean, and is there a level of suffering that they might go through that we would say that's unacceptable? How do we define that suffering? Because suffering is very much determined by what goes on in our own brain, the same situation can cause one person to suffer, truly suffer, and another person to say, yes, it's very painful, but I'm not suffering. I can deal with it. So, who gets to judge that the suffering is enough?* |
|  | 120 | HIV clinician | *I think that, again, offering somebody, who is at that point in their life trajectory, offering them the opportunity for that would be based on them. I guess it depends on what the interventions are exactly. I surely would not want to offer something that may be problematic, that may cause pain, that may make them conceptualize that their death isn't as imminent as they're thinking that it is.* |
|  | 101 | Researcher | *I can imagine a situation where there's considerable uncertainty about the time course of someone's disease where, if one were thinking that they might die within a week, and I have an intervention that it would take me a month or more to get a read on whether the intervention was effective. I don't think it's proper to even offer a participant that, because there's a high likelihood that they would die before any information with gains that has no benefit to the participant and no benefit to society.* |
|  | 109 | Researcher | *Yes, I think it would be ethical. I think because the participants in the program who would have these interventions would be well informed and would understand all the different aspects of it* |
|  | 110 | Researcher | *For me, I would say that depends on the interventions.* |
| Ensuring participants’ understanding of the risks | 115 | HIV clinician | *Yes, ethical, as long as I think a lot of the things we talked about are in place, vetted by insiders, a really good IRB review. And then patients really, really understanding the implications of their choices to join a study like that.* |
|  | 103 | Researcher | *As long as people are well informed of what they are doing and as long as the intervention are reasonable, of course, it's totally ethical.* |
|  | 105 | Researcher | *Because anybody can be offered a research intervention, and if they are informed about their decision and consent to it, they should participate. We all die. So not performing research on dying people seems like we're just leaving out the population.* |
|  | 108 | Researcher | *Because I believe that when our consent form process or IRB process, the HIPAA consent, the Consent to Act, the Bill of Rights, all of that, and the assessment informed consent, all of that is to ensure that we are following the appropriate guidelines in obtaining consent and ensuring that the participant understands what it is that they're volunteering to be involved in.* |
|  | 109 | Researcher | *In this particular instance, interventions at the end of life, I think they would be more informed or just more engaged in the process.* |
|  | 112 | Researcher | *Provided that all the checks and balances are instituted and that they consent and the IRB sign off? Yes. I think you would obviously want to have consent from the individuals who are participating in the study. They need to be informed in I think a rigorous way of what they're about to be involved in, and what they're about to embark on, and I think they would need to be made aware of the treatment options for both their metastatic disease and for their HIV and the pros and cons of continuing with therapy or not continuing with therapy in either case.* |
|  | 118 | Researcher | *We would assure that it's ethical by our moral code, which is adjudicated by panels that we trust, and we call those Institution Review Panels. I think it's as simple as that.* |
| Including community input in trial design | 110 | Researcher | *I think it's important as researcher that we be attentive and listen to what the community is ready to do because I'm constantly surprised that they are ahead of us.* |
|  | 118 | Researcher | *I think it's important, though, to have the community involvement. You get into, I don't want to say problems, but I think sometimes they stray from the real understanding that a moral code is relative to the community in which you're engaged.* |
| Respecting and honoring PWH who choose to participate in interventional HIV cure-related research at the EOL | 113 | Researcher | *Participants that work with us in studies, we are partners, and we engage with them. We honor their ... the time and also, in some cases, the pieces of their bodies that they give to the research team and biopsies and blood samples, all of that that people give. We always want to honor their contributions, making sure they have the biggest impact. But it feels to me even greater at the end of life because it's such a ... I don't know. I would feel that weight more, so I would just make all the more effort to make sure that the work that's being done is important.* |
|  | 114 | HIV clinician | *But really these individuals, it would be nice for their contribution to be acknowledged somehow. I don't know how that would be. But the role that they would be playing by submitting themselves to higher risk interventions for the benefit of society, would need to be appropriately acknowledged, I think, and they need to be held in the highest regard.* |
| **Maximizing Benefits of Testing Interventions at the EOL** | | | |
| Perceived societal benefits of scientific knowledge generation | 104 | Community member | *[C]ertain societal benefits… finding a cure for HIV would benefit the world immeasurably.* |
|  | FG-3 | Participant | *[T]he obvious benefit is that, you know, we discover something new or that works or that's a breakthrough, doing the intervention.* |
|  | 114 | HIV clinician | *Going back to that term of great impact, for society would, it would create knowledge of great impact. The type of knowledge that could be generated from such a study would be very important.* |
|  | 120 | HIV clinician | *There could be some very good science and medicine that comes out of this.* |
|  | 101 | Researcher | *The main advantages are potential gains in knowledge. One of the ones we are most interested in in the Last Gift study would be some new information about the HIV reservoirs that might potentially lead to improved treatments and cure strategies.* |
|  | 103 | Researcher | *[S]o penetration in tissue and in cerebral spinal fluid and brain in particular. We don't know if some of the latency reversing agent have penetrate in the brain. And if there are any side effects in the brain related to these interventions.* |
|  | 109 | Researcher | *Then the second part is on the scientific side, knowing where the reservoirs are, we can't do in someone who is alive. But, when we do a rapid autopsy we're getting all those specimens, so for the first time we can really look and see, did this intervention reach into someone's kidney or someone's liver or someone's spleen? So, this particular Last Gift model allows people a deeper view of those deep tissues where HIV does live.* |
|  | 110 | Researcher | *[I]t would provide information, key information about the effect of some approach like dosing, whether you're about to detect something that would then help in developing clinical trials that would have the potential to work. You would have key information about the approach, but I think the gain would be mostly scientific.* |
|  | 113 | Researcher | *Typically, that's studied in animal models, but there's often a problem with animal models, that they may not fully recapitulate what's actually happening in people. So, there may be a meaningful contribution.* |
|  | 116 | Researcher | *I'm thinking about the scientific ones, it's as simple as that. Then I think it's the speed of getting those. If somebody is at the end of life situation and there are modalities around it, you could get some very clear answers and quickly. Maybe, rule out possibilities.* |
|  | 116 | Researcher | *I also want to know whether the cells that are making that virus are undergoing clonal expansion. In that regard, this is where the Last Gift and then the autopsies can be very various because you'll see that distribution of that virus.* |
|  | 118 | Researcher | *And you're asking for, let's say, pharmacokinetics, or some other, well, primarily pharmacokinetic and pharmacodynamic analogies of the autopsy. Then, in the autopsy, you see the distribution of that agent.* |
|  | 119 | Researcher | *I think a large one is scientific. You'd have a much shorter time to answering critical questions about efficacy in a much smaller sample size, which in turn would potentially have a societal benefit if you had a much better treatment. To the same extent, even if you had tested something that didn't work, you could potentially answer the question faster and expose a smaller number of people to an ineffective treatment modality.* |
| Decreased likelihood of long-term harm | 111 | HIV clinician | *So those sorts of concerns are pretty much nil if you're already at the end of life because you don't have to worry about things 10, 15, 20 years down the line. Things that I think we are considering now in doing some of those studies in younger persons, or persons who are not at the end of their life.* |
|  | 114 | HIV clinician | *But in end-of-life studies, we can perhaps test some interventions that would not be possible otherwise. Because the potential impact from this field is that these interventions could maybe be higher risk than would be for the normal population.* |
|  | 115 | HIV clinician | *I mean, just using some agents that we are hesitant to use in people whose lives are ahead of themselves and could potentially be dangerous. The benefits to society are huge if we can learn what we're hoping to learn in people who've said, "I'm okay and I'm starting to check out so do what you need to do."* |
|  | 120 | HIV clinician | *At end of life, it's not like you're worrying about the intervention being... I mean, we don't want it to be painful, but you're not worried about it being something that might harm them necessarily because they're already there.* |
|  | 103 | Researcher | *I think that these people might be willing to take some increased risk because they don't have to worry so much about side effects of the interventions because people are at the end of life.* |
|  | 107 | Researcher | *These are people that if there are side effects or if there are permanent issues like with whatever organs, tissues, whatever, there would be less of a life that's impacted. And I think that would be the clear benefit.* |
|  | 113 | Researcher | *But something where there is concern about, say, a future harm years down the road, it may not be as much of a concern for someone who knows that they're going to die soon. A long-term toxicity of a medication, for example, may not be relevant in someone who has a shorter life expectancy.* |
| Perceived psychosocial and personal benefits to the participants | 106 | Researcher | *If they get gratification from providing information, then that is a potential benefit, right?* |
|  | 114 | HIV clinician | *Well, personal, even though the individual is at end-of-life, they might get a sense of fulfillment from contributing to greater knowledge about HIV.* |
|  | 115 | HIV clinician | *I think the altruism and giving back to a research community that probably has benefited them along the way. And paving way for other people in the future.* |
|  | 108 | Researcher | *So it would just be really personal…personal satisfaction.* |
|  | 108 | Researcher | *If there is a next of kin involved and there are four family members and, or one or more family members, they get to see their loved one also do something for the benefit of others. And they have a great deal of love and pride and respect for that being done.* |
|  | 109 | Researcher | *If I was at the end of my life and I had an opportunity to give back to the research, so I could help people learn something about the reservoir and how to purge it, then I'd be more willing to take a greater risk in those research efforts. The opportunity to give back in the setting where I have a lot less to lose or a lot more willing to risk my health.* |
| **Minimizing Risks of Testing Interventions at the EOL** | | | |
| Perceived risk of increasing suffering or decreasing QOL of participants | FG-1 | Participant | *I guess my only concern is that any of these modalities would cause any more suffering than is necessary.* |
|  | FG-1 | Participant | *[A]t the end of life, if someone's immune response will be weakened to the point where they wouldn't see as many of these cytokine storms and things like that.* |
|  | FG-3 | Participant | *We're making their end-of-life uncomfortable, putting them in a position where they're in pain, or something like that would definitely be a risk.* |
|  | 115 | HIV clinician | *It's the side effects that a person could incur as a result of this. I think it has to be really front and center for people.* |
|  | 115 | HIV clinician | *I think just the discomfort of something happening with an unknown intervention, and even something as little as I mean, I shouldn't say “as little as,” but just a side effect that's really, it's not maybe super bothersome for someone, but it's persistent and they can't get rid of it.* |
|  | 120 | HIV clinician | *Well, we surely don't want anyone to be in pain. We want that path, that transition, to be as pure and clear as possible for them.* |
|  | 101 | Researcher | *Harm to the participant.* |
|  | 103 | Researcher | *I think that the main risk that we will have to bring up is that some even further abuse the lifespan of the person, of the participant. And of course, we need to be very clear with the participant about that. And too, there might be some side effect, which will make the quality of life worse.* |
|  | 105 | Researcher | *[W]hat if we thought somebody was at end of life, we gave them a treatment that we thought would cure their HIV it did or didn't cure their HIV but for whatever reason, they lived much longer but now with some comorbidity or some side effect of the treatment, a neuropathy or gastrointestinal problem? If you manipulate that you are risking a lot of collateral damage, and then the chance that somebody has increased suffering.* |
|  | 107 | Researcher | *I think trying to convey that to them that, yes, like your overall long term side effects will be less because you're at the end of life, you still may lose quality of life or time.* |
|  | 108 | Researcher | *I would presume that pain, shortening of life, discomfort, fear, anxiety, depression, earlier death.* |
|  | 108 | Researcher | *I would also say that from the next of kin point of view, it could affect the next of kin in some way too. It could increase their anxiety; it could depress them. They could potentially have regrets that if an individual suffered, their loved one suffered who was participating.* |
|  | 109 | Researcher | *It always depends on the intervention. Some interventions are more risky than others. They might also be uncomfortable or make their last moments in life not pleasant. More pain or discomfort associated with whatever intervention that there is at the end of the life, more than what their normal disease process is. Those are the big risks.* |
|  | 110 | Researcher | *[I]f there is any reaction that has not been described for these drugs and that could impact the quality of life of this individual for the remaining time of their life… I think we should reconsider continuing.* |
|  | 113 | Researcher | *Well, I think that anything that increases discomfort, makes it more difficult for people to have meaningful time with their loved ones.* |
| Perceived risk of an intervention hastening a participant’s death | 114 | HIV clinician | *[I]f the intervention perhaps accelerates a participant's death, then that would be bad.* |
|  | 107 | Researcher | *I think the obvious risk is they might die faster.* |
|  | 112 | Researcher | *It’s possible that in some cases, testing interventions in these patients, the HIV could exacerbate their metastatic disease and lead to an accelerated decompensation.* |
| Perceived risk of negative public perceptions of interventional HIV cure-related research at the EOL | 119 | Researcher | *As we say, it's [death] a potentially expected outcome, but it could compromise the program. So, I think you'd have to think very carefully about the agents that you tested and how you went about it.* |
| **Ensuring Acceptable Benefit/Risk Profiles for Testing Interventions at the EOL** | | | |
| Ensuring interventions have solid pre-clinical data and have been vetted at multiple levels | 103 | Researcher | *[W]e have to trust that investigator will only test intervention that are really already been vetted at multiple levels and that they don't use people as guinea pigs, of course. I was thinking something that has solid preclinical data will also, for me, will be vetted at multiple levels, like solid animal data and in vitro data that we know it's the best possible intervention that we can test at this point.* |
|  | 105 | Researcher | *[C]alculating what percentage of people might have the side effect and how that would affect however long they have is part of the risk calculation. So, we think 30% of people could have a neuropathy and in 30% of those people, so 10% overall that neuropathy would be classified as grade three or four severe debilitating. And so therefore your risk going for the next six months is 10%, that you'll increase your suffering at the end of life. But if we can figure out the make of the next step in curing HIV, we could potentially prevent hundreds of thousands of cases of HIV associated neuropathy in the 35 million people living with HIV in the world.* |
|  | 106 | Researcher | *[Y]ou need ethical concern investigators, you need a peer-reviewed protocol, and you need informed consent.* |
|  | 107 | Researcher | *You could start with a lower dose maybe, but I don't know if that would be helpful.* |
|  | 117 | Researcher | *I think if I had to do an intervention in someone at the end of his life, I would probably try drugs that have already been tested in other people before … and make sure that what I'm going to do is not going to induce pain.* |
|  | 119 | Researcher | *I would probably say what I already said, which is a very slow and careful, deliberate consideration about the compounds to be tested.* |
| Creating multi-disciplinary research teams | 104 | Community member | *I think you having the socio-behavioral components integrated into the whole process is key. Doing that start to finish and having it be part of the process and not as an afterthought … it needs to be really part of the DNA of the whole project for it to work well.* |
|  | 111 | HIV clinician | *I think the ACTG model where they have groups that review proposals, that are a mixture of researchers, basic science researchers, clinicians, and community members, is probably the best way to go. I think that that could be a nice way to balance out the risks and the benefits of studies like that. Because it really does capture a full perspective. Certainly, having an ethicist on board in a group like that would also be of extreme value. Again, because ethicists think differently than the rest of us.* |
|  | 108 | Researcher | *We kind of all do sort of a post-visit review of what happened, what could we improve. We have a list of ethical questions or medical questions or consent questions, and we always refine, refine, refine. And we bring back that information to our community advisory board members. We discuss it with other individuals in the field and get their feedback on what their experiences have been. And I think that that continual feedback loop, sharing, being open, revising consents, we do focus groups with next of kin, and I think that that is like very important and we keep it, our wheel of information, continually open and moving. And we're advancing.* |
|  | 110 | Researcher | *I think we need to raise the bar a little bit higher and it comes back to this question of having people knowledgeable that have thought about this question from an ethics point of view on the active committees to really determine whether this is a proposal that is acceptable.* |
|  | 119 | Researcher | *It really ought to be a very public discussion so that the interventions that have greatest potential for benefit with the relatively lowest risk of participant harm. So, a careful public deliberation.* |
| Ensuring research protocols are clearly explained to the participant and consent is revisited throughout the study | 114 | HIV clinician | *I think the investigators need to make it very clear from the outset, the type of risk that is associated with this study and use very clear language. Avoid scientific terms.* |
|  | 115 | HIV clinician | *As long as you're very clearly explaining it to them, even if there is a lot of risk, as long as it's presented to a patient very clearly what that is and what that might look like, I don't think that that's unethical.* |
|  | 115 | HIV clinician | *I would just say a very high level person should be doing the consenting and probably someone who's connected in some way to clinical care or has experience in the clinical care domain.* |
|  | 103 | Researcher | *Informed consent, to me, is the key … making sure that people always know what they're going into and making sure that they know exactly what are the side effects or the risks of the intervention.* |
|  | 107 | Researcher | *I think that there would just have to be some way to really assess their overall understanding. I think that would be helpful just really having almost like a checklist so that they make sure... It's like you teach someone and then they reteach you back.* |
| ***Perceptions of Unacceptable Risk*** | | | |
| Recognizing the participant’s autonomy when deciding what constitutes unacceptable risk | 102 | Bioethicist | *I just thought of another dimension to this, which is to speak, to think in terms of relative risk. In this case, relative suffering. If somebody is suffering independently because of their terminal illness, perhaps in an untreatable way. And now we have some level of suffering on top of that, that is potentially treatable. Now the question is, how much more is that suffering, is the relative change in suffering too much as opposed to some absolute level? Because they're already are suffering, the absolute level of suffering.* |
|  | 104 | Community member | *That would require some really careful advance planning in terms of when, once it is over do they want to linger, do you withhold food and water and things like that to be able to then allow them to be able to die naturally? Because they can't, of course, make their own decisions to enact the end of life. But I think there are a lot of kind of permutations there that need to be explored in terms of how you would make that happen and let people fully participate in a way that they want to.* |
|  | FG-1 | Participant | *That's gonna be different for everybody. I mean, there's some people that are afraid of needles, there's others that aren't. Some people just absolutely can't stand vomiting and they'd rather have diarrhea, or they'd rather go through flu-like symptoms without vomiting and vice versa.* |
|  | FG-3 | Participant | *I think it really would be up to that patient and to a point the patient's care team, you know, I think that they should be involving their care team. And if they're gonna experiment with the new intervention, they should at least have the right or the ability to run it past their primary care team to see, you know, how they feel about it or how they think it's gonna affect the patient and kind of make a decision from there.* |
|  | 108 | Researcher | *As far as how painful or not painful or how severe or not, that really pretty much is an individual kind of a choice. They would have to decide that on their own, I can't.* |
|  | 109 | Researcher | *Because some people might be very much wanting to participate in science, and even if it meant that they lose an extra month of their life, they think that that might be worth it.* |
| Causing undue pain and suffering | FG-1 | Participant | *I'm just concerned that, you know, at the end of life I don't really want to suffer any more than I have to.* |
|  | FG-2 | Participant | *I would say anything that puts you in pain or makes you more uncomfortable would be something I would think twice about before consenting to.* |
|  | 111 | HIV clinician | *I think... significant suffering, whether it be pain, or shortness of breath, severe nausea and diarrhea, if an intervention were associated with significant suffering along those lines, I would be very uncomfortable recommending it at the end of life. Again, because it's a tough time already, and I wouldn't want to additionally burden a participant with symptoms that they're voluntarily ascribing to.* |
|  | 114 | HIV clinician | *If there would be unnecessary pain or discomfort to the participant. These are individuals that are end-of-life…. Something that would cause pain or discomfort, embarrassment, anxiety to the participant or to the participant's family or caregivers.* |
|  | 115 | HIV clinician | *I mean, something that just made someone feel terrible all the time. I keep coming back to nausea, because I think for me, that's the thing I would not deal with. In studies like this, if you know that there's a really significant side effect, but the side effect is manageable with either pain medications or, anti-emetics or something else.* |
|  | 120 | HIV clinician | *Of course, too much risk would be anything that would be painful or harmful. I'm not sure that there's, other than that, a blanket answer.* |
|  | 105 | Researcher | *I would worry much more about living a long life with suffering than dying quickly.* |
|  | 106 | Researcher | *Something that caused undue pain or discomfort or could have a negative health consequence. Drug toxicity, for example.* |
|  | 107 | Researcher | *A known issue that was really going to impact their quality of life, I think that would be too much.* |
|  | 108 | Researcher | *I would want to have very good pain control, personally.* |
|  | 108 | Researcher | *And also I think we're obligated to remind them that if it's too much pain, too much anxiety, too much depression, they're volunteers. They do have the right to withdraw at any time, and also that their next of kin understand that too.* |
| Rapidly hastening death | 101 | Researcher | *The way I would think about this is the same way that I would think about cancer chemotherapy with the attendant risk of death. We look at, well, what's the likelihood that an individual will die from their primary disease? What is the potential course of their disease if they go untreated? What alternative options are there for treatment? How many of those options have they exhausted? And make it very explicit to the participant that the intervention may hasten their death.* |
| Causing unintended effects that lead to prolonged pain and suffering | 115 | HIV clinician | *But leaving someone with some unalterable change in them, I don't know, memory loss, something that was really profound.* |
|  | 105 | Researcher | *Mucking with the immune system can yield so many pleiotropic effects. So the one I would worry about is we actually cured them of their underlying HIV and/or their underlying malignancy, whatever was going to kill them because we gave them CAR T cells against HIV and there was an off target effect that completely obliterated their pancreatic cancer, but also knocked out their splenic nerve and a couple of other nerves. And so now, they're paralyzed and can't move their bowels and are going to live for forever.* |
|  | 107 | Researcher | *[If] we knew that it was going to cause them to like go blind or have some permanent disability that would further impact their life I think as researchers that that would be too far.* |
|  | 112 | Researcher | *[S]ome proliferative agent for example, that increases cell proliferation, even if it were theoretically able to ... or theoretically had the predictive capacity to lower HIV, but would exacerbate the cancer, I would consider that to be just off limits.* |
| **Minimizing Participant Burdens at the EOL** | | | |
| Designing protocols that are flexible and adaptable to each participant | 104 | Community member | *[I]t's their choice and the study makes it possible for them to opt out if they're having a bad day or whatever.* |
|  | FG-2 | Participant | *Being flexible. . . flexible with visits.* |
|  | 120 | HIV clinician | *Just tailoring it to the person, I think, is going to be important. I think if researchers are able to stay nimble with that, I think that that would be the very best thing.* |
|  | 105 | Researcher | *Let's not forget that many people at the end of life can have already a million doctor's visits or clinic visits or whatnot for chemo and whatever it is that they're going through. So, to some extent, a few extra visits may not feel like very much, but I think we just have to keep them as simple as possible and just try to remove those barriers.* |
|  | 107 | Researcher | *I think maybe offering a study with more options. So maybe really think about what's absolutely necessary and then have everything else kind of be optional, so that they don't feel like they have to take on more burden than they can tolerate.* |
|  | 107 | Researcher | *I think if you're designing a study that's saying we absolutely need blood drawn at week 12 [then] I think you're creating a barrier to your research from the beginning, because if they are not stable enough to do something at that point, whether that's blood or an assessment of some other sort and then you've put a lot of emphasis on a timeframe.* |
|  | 116 | Researcher | *The protocol would want X but, based on the condition of the patient, you can't have X. You can have 50% of X and that's still OK. As long as you can get a reasonable research outcome from it, it justifies the draw.* |
|  | 116 | Researcher | *I think those things are going to tend to be individualized because of the circumstances, somebody's going to have and the outcome at the end of life.* |
|  | 117 | Researcher | *It's partly also participant dependent, and it depends on which disease they're suffering from.* |
|  | 119 | Researcher | *Flexibility within the protocol… that requires a lot more work. [F]lexibility is the answer.* |
|  | 119 | Researcher | *We modify the protocol a little bit to be a bit more case by case. So, it really does require quite a bit more flexibility within the constraints of the protocol.* |
| Enabling research teams to travel to the participant | 114 | HIV clinician | *Going mobile, going to the individuals using telehealth or video visits or phone communications as much as possible, blood draws to pull them or whatever.* |
|  | 115 | HIV clinician | *[T]hat study coordinators go to participants homes. I obviously think the more that you can do that, the better.* |
|  | 120 | HIV clinician | *In my mind, I would think that going to them would be perhaps the way that would help them be least bothered, have their life be the least affected.* |
|  | 103 | Researcher | *Well we could, for example, offer to go at their house to perform some of the procedures. For example, blood draw can be easily performed at their place. This is what we do also with the Last Gift people to try to minimize transportation of people, for example.* |
|  | 105 | Researcher | *[W]e make it as smooth as possible for Last Gift; we're willing to do home visits.* |
|  | 108 | Researcher | *What we've done is we have, when it is possible, we've offered to go to the individual's home to do the blood draws.* |
|  | 113 | Researcher | *If they're in a hospice, is there a way that the research team can go to them to minimize the ... or that a study nurse could visit them in the hospice for some things, minimize their need to go back and forth. Anything that could be done to make things easier for the participant ... It's hard to talk in hypotheticals, but just a genuine effort by the research team.* |
|  | 118 | Researcher | *Certainly, with telemedicine, now, you could see them at home.* |
| Ensuring blood draws do not require participant travel | FG-1 | Participant | *Is it possible for labs to send a mobile lab to the patient's house to do draw rather than trying to transport the patient in the middle of all of this? Is that even a possibility that a mobile lab could go to the patient's home to do all the labs, all the blood draws?* |
|  | 112 | Researcher | *I don't think a hospital visit is necessarily needed for a blood draw. I suspect it could be a home visit as well or a hospice visit* |
|  | 118 | Researcher | *You can draw blood at home. You can have a home phlebotomy visit.* |
| Providing transportation for participants/caregivers | 104 | Community member | *You [study team] provide the transportation.* |
|  | FG-3 | Participant | *I would suggest having a car service that you know, you can depend on as opposed to say, Uber or Lyft.* |
|  | 115 | HIV clinician | *Obviously covering any costs associated with transportation, which I think is an obvious one.* |
|  | 108 | Researcher | *We've offered in the past transportation of individuals to our clinic for blood draws or for MRIs or for neuropsychological assessments. And we also have provided transportation compensation in the form of gas money, or by providing a taxi or town car, depending on the distance that they live away from the facility that they're being seen at.* |
| Incentivizing participants for their time and effort (minimizing financial burdens) | 114 | HIV clinician | *[P]retty much compensating for any other hidden costs to the patient that I can't anticipate right now.* |
|  | 108 | Researcher | *We’ve compensated them.* |
| **Integrating Considerations for Next-of-Kin/Loved Ones** | | | |
| Engaging NOK/loved ones early in the research process | 104 | Community member | *[I]nvolving the family, next to kin, partners that sort of thing, so that everybody's on the same page and really understands all the ins and outs of what that means throughout the whole process* |
|  | 104 | Community member | *I think we need to do the same process that we've been doing and really engage them in this. I think really kind of walking people through that process so that they understand [and] I think [that] can alleviate that fear and anxiety that people might experience to know that that's what's done in a really good hospice, nurse, or staff member can help with that process.* |
|  | FG-1 | Participant | *I'm wondering if it's possible to retain from each family, contact information for one family member that in the event there is a groundbreaking revelation that comes from this study that perhaps that could be sent to a family member. That may give them some comfort or understanding after the fact as to why their loved one, family member participated.* |
|  | FG-2 | Participant | *I think we just need to be aware that those exist and even if an individual participant is interested, their friends or family members might still have that mistrust and that could complicate things.* |
|  | FG-3 | Participant | *I would like it to be clean and socially acceptable for them to be involved and have, maybe less some, some ideas about how we could, you know, lay it down, how we could talk to our family about it.* |
|  | FG-3 | Participant | *[L]ike asking the patient who their support system is. Because a lot of us have families of choice versus really involved biological families.* |
|  | 120 | HIV clinician | *Well, it's important that they [NOK/loved ones] be on board as well. It's important that they have a clear understanding of what's happening. I think our culture does a terrible job with death.* |
|  | 105 | Researcher | *I think in my experience with last gift, the most important thing to convey to the next of kin and loved ones, is how much the participant believes in this research. If you as a participant want your body given for a rapid autopsy, and the study has explained to you why they want it and you've agreed to it, it's incredibly powerful to tell their family, this is what they want. And obviously, let them hear ideally from their loved one, the participant, because you can win the family over the next of kin over by just letting them hear the participants words.* |
|  | 106 | Researcher | *It's always good if they have a support system that agrees with the decision making.* |
|  | 108 | Researcher | *It reduces a lot of anxiety for folks and it's an interesting dynamic, the participant/significant other communication, not all of our participants have shared with their next of kin that they were even at the end-of-life.* |
|  | 108 | Researcher | *It's important that they understand that there is some benefit and risk. It's important they understand by encouraging them to speak with their loved ones about the reasons why and the feelings why they want to do this, and for them to understand that their participant has the right to continue in the study, we hope that the next of kin would support that, they also have the right to withdraw from the study, and we hope that the next of kin would support that too.* |
|  | 108 | Researcher | *That's also one of the reasons why I went from having meetings with one-on-one with the participant to including the next of kin with the participant's approval, because I thought it was very important that the next of kin also be aware of what's going on and why they're participating and what actually is going to happen and what's not going to happen.* |
|  | 109 | Researcher | *I think that the loved ones and family have to be considered in all those things. That takes place with an open dialogue first with the potential participant and then the participant with their discussion with their family and loved ones.* |
|  | 116 | Researcher | *Well I guess, presumably, they would be involved in all of the discussions or at least I think that would be really, really important.* |
|  | 118 | Researcher | *The education of the next of kin is the most critical thing. So, they have to have bought into this whole idea, so that when the end comes, they're really, in a way, relieved to know that their last effort will be to notify the system, so that the team comes in for the autopsy.* |
| Recognizing participant’s autonomy in deciding whether and whom to include as NOK/loved ones | 110 | Researcher | *When someone suffers from terminal cancer and they are proposed [an] experimental approach which probably in some cases will not extend their life, are more to learn more about it than, we're not asking permission to, there is a discussion with the family and everything, but at the end, it's the patient that accept or not.* |
|  | 119 | Researcher | *I want them to be involved, but I wouldn't exclude a participant because their next of kin is not on board. I think they need to understand that this may be harder for you because your next of kin is not on board … but at the end of the day, it is their decision. So, it'll be harder for you if your next of kin, if your partner, your spouse, your whoever is not engaged in the process.* |
| Recognizing the emotional nature of EOL research and interpersonal dynamics | FG-3 | Participant | *Emotional support. Sometimes the patients, you know, the patient themselves don't feel like explaining everything in detail from family. It's better to refer that to somebody else, a professional, who can really take care of that part of the emotional support and the explaining of what's happening so that the patient could be really left out of that part if they choose to.* |
|  | 111 | HIV clinician | *I think that that may be difficult for some partners. Some partners may not be ready to accept that it's the end of life…. So I can see that there could be quite a significant barrier there, if the partner and the participant's wishes are not totally aligned* |
|  | 112 | Researcher | *That you have to follow the more conservative person and you can't go with the intervention because you're affecting the partner's life as well potentially with an intervention that could require a lot of caregiving, or even an emotional caregiving if it causes pain.*  *I think you're going to need both parties. It's not an “or,” it's an “and.” Both parties to agree* |
| Support for NOK/loved ones providing informed consent | 104 | Community member | *But I think having them document that, yes, I understand what's going on, I support my partner's wishes. That sort of thing could be a valuable conversation to have, whether or not a signature on a piece of paper means much. But I think the process of getting to that point would be very valuable for all concerned.* |
|  | 120 | HIV clinician | *I'm thinking, actually, as we're talking about it, that they probably should for several reasons. One, you want them informed. But two, their feedback could be incredibly invaluable, moving forward. To make this research as palatable for everybody, I think that having them guide future interventions is going to be a really important key to that process.* |
|  | 107 | Researcher | *I guess it makes sense the more I kind of talk about it that they would sign a consent just because there's no disagreement then later if they get upset. And I think it's not unexpected that if something bad happens that they might be upset. So, I suppose it makes sense.* |
| Opposition to NOK/loved ones providing informed consent | 111 | HIV clinician | *I don't think they should be consented; I think they should be involved in the consent process.* |
|  | 114 | HIV clinician | *No. I don't think so. This is very personal and private. I think of an individual as not have the capacity to make this decision, then no one should sign it for them.* |
|  | 103 | Researcher | *I think that having the next of kin provide informed consent might be almost a little bit infantilizing the patient himself. I think it's really important that we trust the process and that the patient can make the informed decision.* |
|  | 108 | Researcher | *I also don't want to hold back participation because we do have many participants or potential participants that have no one else. So, if there is no one else, we don't want that to hold them back from participating because they have no one else to confer with or to give additional consent to.* |
| ***Considerations for Testing Specific HIV Cure-Related Research Strategies*** | | | |
| **Perceptions and Safeguards around Testing Latency-Reversing Agents in the EOL Translational Model** | | | |
| Support for testing LRAs in the EOL translational model | FG-1 | Participant | *So, I think short answer, yes. I'm shocked that you didn't get the lock and block in there anywhere.* |
|  | 107 | Researcher | *I've done studies with those and there's lots of different research from like benign to not so benign with those. But yeah, I could see that as being fine.* |
|  | 108 | Researcher | *I think that's a good one.* |
|  | 110 | Researcher | *I think that this type of approach would be acceptable. They would be acceptable, because … lot[s] of these drugs are repurposed.* |
|  | 117 | Researcher | *I'm not a big specialist of the clinical effects of LRAs, but my understanding is that most of their areas that they’ve been tested so far in the clinics, I would say relatively safe.* |
| Reservations regarding efficacy of LRAs | 114 | HIV clinician | *Yes. With the appropriate precautions. Yes, I think we can… The only thing I would say is, to try an agent that has as much robust preclinical data about safety and efficacy.* |
|  | 105 | Researcher | *I don't personally think latency reversing agents are probably the way forward or if they are they're going to be part of a cocktail, but they should be tried.* |
|  | 119 | Researcher | *I don't think either of those agents is ready for prime time, and the ones that we have are probably marginally effective.* |
| Considerations depend on specific LRAs | 105 | Researcher | *This is all gonna have to be spelled out based on animal models, and we tried this drug and these animals, and nobody lost weight or nobody died early, but a few people had fevers or few animals had fevers. It just needs to be balanced and explained.* |
|  | 112 | Researcher | *Yeah. It depends on the latency reversing agent to be frank with you. If it’s a chromatin based histone deacetylase or something like that, no. Just no. For a long time, I’ve held that view. I was an outlier until recently, and then it’s become very clear these things aren’t working.* |
| **Perceptions and Safeguards around Testing Immune-Based Interventions in the EOL Translational Research Model** | | | |
| Suggestions for immune-based interventions to be tested | 106 | Researcher | *One thing that strikes me as potentially interesting is the expression vectors of antibodies, where I don’t think there’s any risk and it may be a good population to test initially.* |
|  | 107 | Researcher | *[M]ost of the broadly neutralizing antibodies I’ve dealt with have been actually very safe, … so the antibody therapy I feel like is pretty safe.* |
|  | 110 | Researcher | *No, I saw that you were including CAR T-Cell, so I would put CAR T-Cells in the same type of approaches where I’m a little bit cautious about this and the effects that they might do in an individual at the end of life.* |
|  | 110 | Researcher | *I think the infusion of neutralizing antibody has less risks, for example, than approaches that try to boost immune system to make the immune system overreact.* |
| Greater monitoring required as an additional safeguard | 111 | HIV clinician | *I think... having pretty close monitoring…* |
|  | 105 | Researcher | *Just whatever you do for chemo, the one caveat right with antibodies, you can get serum sickness, so you just need to have a specific monitoring parameter for that and be ready to reverse it with steroids.* |
| ***Perceptions and Safeguards around Testing Cell and Gene Therapy Approaches in the EOL Translational Research Model*** | | | |
| Support for testing CGT approaches in the EOL translational model | 111 | HIV clinician | *I don’t have any problems with that. I think... it’s the delivery that’s the issue … how do we actually get these interventions to the latently affected cells? And I think that cell and gene therapy may be... if it’s paired with some novel delivery, could be very effective.* |
|  | 113 | Researcher | *Yeah, I think those would be reasonable to test… There’s no reason why someone at the end of life should not be able to participate in that type of research. So, I think that’s fine. I mean, because all these interventions are being thought of for people who are not at the end of life, I would just draw the line on something that is expected to cause a lot of toxicity in the moment and discomfort in the moment.* |
|  | 116 | Researcher | *Yes. Essentially, for the same reasons. We have reasonable evidence that these therapies can actually have the potential to affect reservoirs in vivo.* |
|  | 118 | Researcher | *[T]he potential one, where you use a lentiviral, a direct injection lentivirus… It could be direct injection, in general, of different genetic strategies, might be a perfect population in this group.* |
|  | 119 | Researcher | *[T]his kind of therapy, I think, has the potential to move the needle a lot.* |
| **Perceptions around Testing Stem Cell Transplants in the EOL Translational Research Model** | | | |
| Opposition to testing stem cell transplants at the EOL because of high risk profile and significant discomfort caused to participants | 106 | Researcher | *Which have a mortality risks of 20%... I’m not sure I would want to participate with conducting a study like that.* |
|  | 109 | Researcher | *[S]tem cell transplants are really dangerous and cause lots of discomfort. I don’t know if they’re dangerous, but they can be and they can cause lots of discomfort to people. So graft-versus-host disease and a greater number of infections, et cetera…* |
|  | 109 | Researcher | *But the thing that I would most worry about is that those people would be really at risk for some of those infections and graft-versus-host disease, et cetera. That would always be worrisome.* |
|  | 112 | Researcher | *Those are complicated. As I understand it, the mortality from a bone marrow transplant is 50% survival for five year or four year period… [I]t’s an incredibly invasive and risky procedure and, whether the patient would survive that, I think needs to be weighed.* |
|  | 113 | Researcher | *Well, a stem cell transplant typically does require conditioning and typically does require quite a bit of discomfort. So, I would not feel comfortable proposing that at the end of life.* |
|  | 116 | Researcher | *Ouch!* |
| Recognizing the potential gain in scientific knowledge may be great yet there may not be enough time to realize the effects of stem cell transplants | 104 | Community member | *Again, it’s all … the scientific feasibility I think should dictate that…And again, with the same caveat, it’s about ensuring they’re comfortable and not in pain, it doesn’t cause you any distress… [S]tem cell transplants or something that there’s time for the actual chimerization to happen, to see if it’s really effective.* |
|  | 109 | Researcher | *Then it would really depend on what exactly sort of stems cells that you’re transplanting. Is it just a transplant of a stem cell that has the CCR5s, such as what was done with Timothy Brown and then others, the London patient. Then we could learn a lot in that process because if we did a transplant, there was engraftment, then we could also look at where all those new T-cells went in the body when we actually did the autopsy.* |
| Recognizing the expense and high risk profile of stem cell transplants significantly hinder the scalability of this approach | 111 | HIV clinician | *It’s really expensive, and usually in order for it to work you do have to put the participant through a pretty intensive round or rounds of chemotherapy, which do cause some suffering. So, I think I’m less favorable about that approach* |
|  | 110 | Researcher | *How this information and the treatment that could be developed through this will be accessible to the entire people that are infected with HIV? I mean, if this will benefit only a minority of people that can pay this type of treatment. The debate here is, I think we should take into account this, how the development of treatment through end of life and the altruism of people that doing this will serve the largest number of people in term of approach to cure HIV, because if this is to be able to cure a minority of people that have the means to pay, I would be completely against it.* |
| **Perceptions and Safeguards around Testing Novel Approaches in the EOL Translational Research Model** | | | |
| Describing TIPs mechanism of action | 109 | Researcher | *How it works is basically for this particular purpose, someone has HIV and you give them a defective type of HIV particle. What that means is that the only time that this detective particle can grow is when HIV is growing. But when HIV grows, it causes this particle to also grow, but out compete HIV. So every time that HIV tries to grow, this other particle eats up all the stuff that lets HIV grow, so capsid, all the different building blocks of HIV. It just can do it faster. So every time that HIV busts out, this particle eats its lunch, that's basically what happens, which is fascinating. If it weren't, then you could give somebody this interference particle and it would hang out and every time that HIV tried to come out, it would just squash it by eating all of its food basically.* |
|  | 112 | Researcher | *The static drugs, and the dynamic living system. And so, many years ago, we had the idea of can we create a therapy that solves this mismatch and mutates and co transmits along with the virus. And that's what we set out to do… [W]e call it a therapeutic interfering particle. It is mobilized by wild type virus. When the wild-type virus infects the cell, this interfering particle competes with the wild type virus, and what you get is the cell becoming a factory to produce the therapeutic, and it spreads to other cells, and lowers viral load.* |
|  | 112 | Researcher | *Whereas the interfering particles that we've developed, can only transmit between infected people, in the high risk populations…* |
| Recognizing the uncertainty of TIPs |  |  | *[W]hether by introducing these particles, we're going to cause some inflammatory response and make things worse because of inflammation, we just don't know. No one knows. There are arguments both ways that this is going to happen and then there are arguments like no, this is not going to happen. And we're just going to have to test and see.* |
| **Perceptions and Safeguards around Analytical Treatment Interruptions (ATIs) in the EOL Translational Research Model** | | | |
| Support for the use of ATIs at the EOL | FG-2 | Participant | *[I]f someone’s a long-term survivor with HIV, they have seen the ups and downs of taking and not taking medicine, so they should know what to expect stopping medication.* |
|  | FG-3 | Participant | *I would be fine with it if I were at the end-of-life… I would ethically be okay with that.* |
|  | FG-3 | Participant | *[I]f I’m coming closer to the end of the life, I wouldn’t mind stopping it, just to see… That wouldn’t bother me. That would be something I’d be willing to do.* |
|  | 105 | Researcher | *I think it’s going to be necessary to prove that you did what you did.* |
|  | 109 | Researcher | *I think that stopping the therapy happens anyway, because of their life, and being able to observe it and learn from it scientifically I think is low hanging fruit.* |
|  | 110 | Researcher | *The analytical treatment interruption, if they’re done within the boundaries of how they are done currently and are done in a safe way and everything, I think that would be acceptable to do it, but again it has to be done within the boundaries of what is acceptable right now.* |
|  | 112 | Researcher | *As I understand, it already happens naturally that many patients come off of their drugs voluntarily because they are informed from their attending physician that the HIV is essentially not going to kill them, it’s the metastatic disease. And so they see no reason to take these drugs. So, based on that, I would say the precedent is there.* |
|  | 113 | Researcher | *I think they’re totally reasonable at the end of life. I think they’re reasonable also in individuals who are not at the end of life with appropriate precautions* |
|  | 116 | Researcher | *Resistance wouldn’t be as big a deal at the end of life either, if you’re that proxy.* |
|  | 117 | Researcher | *I know that in the Last Gift Study people actually interrupted antiretroviral therapy. This is clearly a great opportunity to understand where the virus comes back, where it comes, where it was persisting, and then we have the virus, where the rebound originates.* |
|  | 118 | Researcher | *Nothing will be worse than what they’re facing, anyways.* |
|  | 118 | Researcher | *Well, maybe you’ll get a virus that is resistant. The person’s probably not ever going to go back on therapy, anyways. Maybe you’ll get viral syndrome. You’ll get a flu-like illness. Well, that could be managed.* |
| Recognizing increased risk of HIV transmission and protecting against it | 111 | HIV clinician | *[B]eing very honest with them about the unknown risk. It's hard to talk about that, because it's very possible there's no risk, but we have to review all the potential hypothetical risks regardless of whether or not we think they're real.* |
|  | 115 | HIV clinician | *I think it's the same concept as what I've been saying, which is, as long as the risks are very clearly made to the participants, to me, all is fair.* |
|  | 103 | Researcher | *Absolutely to avoid transmission to other people. So, we need to make sure the patient and their partner are well informed about the fact that when the virus comes back* |
|  | 103 | Researcher | *And then to monitor for symptoms related to acute retroviral syndrome like fever or sore throat or whatever can happen when the virus comes back, and make sure this is acceptable for the patient.* |
| Ensuring close monitoring procedures | 105 | Researcher | *I guess make sure they don't drop their CD4 count too low, I don't know. Just close monitoring* |
|  | 106 | Researcher | *The usual monitoring and mutual understanding about if and when treatment should be resumed.* |
|  | 112 | Researcher | *Yeah, I guess my safeguards will be based on CD4 T-cell counts and AIDS associated diseases… So if they started to manifest Kaposi's sarcoma or Pneumocystis, I would then certainly start them on an antiretroviral therapy regimen on a combination regimen.* |
| ***Additional Considerations for Interventional Research at the EOL*** | | | |
| **Ascertaining Death as a Serious Adverse Event** | | | |
| Ascertaining whether death was caused by participant’s underlying terminal illness or by the intervention | 102 | Bioethicist | *And COVID is a good example of that, where somebody could have other underlying diseases. And if they come down with it, as far as SARS-CoV-2, and then die, is it because of COVID? As a result of SARS-CoV-2 or is it because of their underlying disease? Most probably because of the combination, but we would really say that the thing that pushed them over was the COVID.* |
|  | 102 | Bioethicist | *My understanding of the term serious adverse event is when you have a disease and certain things are expected, that's part of what you expect. And so, if you have a terminal illness, you are expected to die* |
|  | 104 | Community member | *I think it's an expected outcome. I think that it would be important to try to ascertain if it's due to the therapy because of you're trying to translate this into research and healthier volunteers. You obviously don't wanna do something that's going to kill them.* |
|  | 114 | HIV clinician | *Yeah, it is. It might be difficult to ascertain, if death happens, how it is related exactly to the intervention or to the research study itself?* |
|  | 106 | Researcher | *Well, the same criteria as with any adverse event. You say unrelated, a possible, probable, or definite, and you have to make those same sorts of decisions. The sicker the patient is, the more complex that usually is.* |
|  | 112 | Researcher | *Yeah, that's a hard technical question. I thought about it a little. I've never had a good answer, but one way that maybe that it could start to be dealt with was based on meta-analysis of the patient's particular cancer. And I assume that both locally in the hospital that they're being treated in and in the country as a whole there are large data sets of what are the most common causes of death or failures to thrive in individuals with this particular cancer.* |
|  | 116 | Researcher | *I think making that SAE ascertainment is probably pretty important.* |
|  | 117 | Researcher | *I guess many of the doctors involved in this type of research would be a little bit worried about this because that would be important to show that you have not killed a patient by doing what you did.* |
|  | 118 | Researcher | *You deal about it as you would getting hit by a car. It was unrelated to the research, I think, in most cases. That person with the multiple myeloma got a very high calcium level, and then died. It was consistent with his disease, which existed before the treatment started.* |
|  | 118 | Researcher | *[Y]ou could remove death as a dose-limiting toxicity because, it would never be... it's always going to be likely to be unrelated.* |
|  | 119 | Researcher | *Death is an SAE when it is unexpected. So, this is expected.* |
| Reporting all deaths for transparency and ensuring independent review of the cause of death | 111 | HIV clinician | *I think what has to happen is it has to be reviewed, probably by at least two doctors, so that... because I think the distinction has to be made, is it death because of the intervention? Or is it death from natural causes? And those are going to be very difficult to tease out, potentially.* |
|  | 105 | Researcher | *The other thing to do is have some committee that gauges whether the treatment played a significant versus or it vastly increased the risk of the cause of death versus the cause of death was essentially just a mild acceleration of their pancreatic cancer due to research or something.* |
|  | 109 | Researcher | *So how you would have to do it is to have the back and forth with the FDA and how I think we would do it is to explain that that is, quote an end point of this study and it's not unexpected. It will still be classified as an SAE and I don't think that there's anything wrong with that, it's just an expected serious adverse event.* |
|  | 116 | Researcher | *I'm going to report SAE's, and you can say, I want to report all my SAE's at the time of continuing review because death for every one of these is going to be an expected outcome.* |
|  | 116 | Researcher | *Yeah, I think it's got to be reported and reviewed but I don't think it should be stopping the protocol in any way.* |
| **Conducting Research with Participants with Concomitant Conditions at the EOL** | | | |
| Support for testing interventions in participants with concomitant conditions at the EOL | 114 | HIV clinician | *Yeah. I think, why not? I think, again with proper precautions, proper disclosures, full consent, a strong safety protocol, then yeah.* |
|  | 103 | Researcher | *If you select some of the therapies that we use for HIV have already been used for cancer. So why not? If somebody has HIV and cancer and you use anti-PDL-1 or an immune modulatory intervention, for example, then you can see the effect on both HIV and cancer.* |
|  | 103 | Researcher | *HIV and in cancer, probably not a problem.* |
|  | 106 | Researcher | *Yeah, we do that with treatments for opportunistic infections. We monitor both the HIV and the opportunistic infection.* |
|  | 116 | Researcher | *One of our papers describes a patient with HIV, advanced AIDS, and Castleman's, and primary effusion lymphoma, who got pembrolizumab. I'm sort of interested in what's going to happen to the reservoir in that circumstance. Yeah, I think there are circumstances where that's probably the entrée for a lot of these drugs. Certainly, for things like pembro being used as anti-cancer agents. Yeah, I think that's definitely a possibility.* |
|  | 117 | Researcher | *That's actually a question that is already ringing a bell for me, because we did such studies where we had those people were suffering from melanoma, and one of the drugs we give these people are these immune checkpoint blockers to treat their cancer and particularly P1 and anti-CD4. We thought that these antibodies or these human check point blockers could also have an impact on the HIV reservoir.* |
|  | 117 | Researcher | *Cancer is probably the best example. You mentioned CAR T-Cell. It depends. Because for CAR T-Cells, usually you design CAR T-Cells that are specific to either the tumor antigen, or viral antigen, so you can imagine to combine both why not. … That might be an idea for some of the latency reversing agents that have been used in cancer in the past, so that's pretty easy, I guess, to find an indication for those, the anti PD1 anti CD4 is probably one of the best examples we have so far.* |
| Concerns about testing interventions in participants with concomitant conditions at the EOL | 120 | HIV clinician | *I mean, it makes it a little bit more difficult to do the statistical analysis, but I absolutely think that yes* |
|  | 110 | Researcher | *I think we need to really take that [concomitant conditions] into consideration and see if this will have a confounding effect with the type of question that you're asking.* |
|  | 118 | Researcher | *If it is a trial sponsored by NIAID, I don't think you should study ALS, but if it is something that's sponsored by the appropriate institute, then I would say yes.* |
|  | 119 | Researcher | *When you magnify that by saying, "Now we're going to treat two conditions," it amplifies exponentially the number of challenges involved in the research* |
